# Lung Cancer Induces NK Cell Contractility and Cytotoxicity Through Transcription Factor Nuclear Localization

**DOI:** 10.3389/fcell.2022.871326

**Published:** 2022-05-16

**Authors:** Darren Chen Pei Wong, E Hui Clarissa Lee, Junzhi Er, Ivan Yow, Ricky Abdi Gunawan Koean, Owen Ang, Jingwei Xiao, Boon Chuan Low, Jeak Ling Ding

**Affiliations:** ^1^ Department of Biological Sciences, National University of Singapore, Singapore, Singapore; ^2^ Mechanobiology Institute Singapore, National University of Singapore, Singapore, Singapore; ^3^ University Scholars Programme, National University of Singapore, Singapore, Singapore; ^4^ Integrative Sciences and Engineering Programme, National University of Singapore, Singapore, Singapore

**Keywords:** cellular contractility of natural killer (NK) cell, transcription factor shuttling, Eomesdermin (Eomes), non-small cell lung cancer (NSCLC, invasive and non-invasive subtypes), NK-cancer cell interaction, TGFβ disparate role, mechanotransduction in NK cells, actin cytoskeleton

## Abstract

Actomyosin-mediated cellular contractility is highly conserved for mechanotransduction and signalling. While this phenomenon has been observed in adherent cell models, whether/how contractile forces regulate the function of suspension cells like natural killer (NK) cells during cancer surveillance, is unknown. Here, we demonstrated in coculture settings that the evolutionarily conserved NK cell transcription factor, Eomes, undergoes nuclear shuttling during lung cancer cell surveillance. Biophysical and biochemical analyses revealed mechanistic enhancement of NK cell actomyosin-mediated contractility, which is associated with nuclear flattening, thus enabling nuclear entry of Eomes associated with enhanced NK cytotoxicity. We found that NK cells responded to the presumed immunosuppressive TGFβ in the NK-lung cancer coculture medium to sustain its intracellular contractility through myosin light chain phosphorylation, thereby promoting Eomes nuclear localization. Therefore, our results demonstrate that lung cancer cells provoke NK cell contractility as an early phase activation mechanism and that Eomes is a plausible mechano-responsive protein for increased NK cytotoxicity. There is scope for strategic application of actomyosin-mediated contractility modulating drugs *ex vivo,* to reinvigorate NK cells prior to adoptive cancer immunotherapy *in vivo* (177 words).

## Introduction

The innate immune Natural Killer (NK) cells do not require antigen sensitization and are fast-acting first-line responders to pathophysiological conditions of infection and cancer. Nevertheless, the prowess of NK cells is mediated by expression and balance of its activating and inhibitory surface receptors along with transcriptional control of cytotoxic molecules which, altogether, regulate NK cell cytotoxic activity (Brennan et al., 1994; Stabile et al., 2017; Frutoso and Mortier, 2019). The evolutionarily conserved Tbr1 subfamily of T-box transcription factors (TFs) (Papaioannou, 2014), Eomes and T-bet, are the only T-box proteins expressed in cells of hematopoietic origin (Simonetta et al., 2016; Wagner et al., 2020), and are known to regulate of immune cell development and functions.

Earlier studies in murine models purported redundant roles of Eomes and T-bet in CD8 T-cells (Weder et al., 2014; Zhang et al., 2018). However, in the context of NK cells, the study of Eomes and T-bet has been mainly restricted to their roles in the regulation of stage-specific NK cell maturation, with the expression of Eomes being crucial to functional maturation (Simonetta et al., 2016; Wagner et al., 2020; Zhang et al., 2018, 2021). Interestingly, although much focus on NK maturation has been on the balance of expression of Eomes and T-bet (Zhang et al., 2018), information is lacking on how Eomes and T-bet are compartmentalized for NK cells to elicit anticancer activities. Understanding the molecular and/or biophysical mechanisms and triggers that influence Eomes and T-bet localization is relevant since previous studies in CD8 T-cells highlighted preferential nuclear localization of Eomes or T-bet based on either CD8 exhausted (McLane et al., 2021) or memory T-cells (McLane et al., 2013). These findings indicate a context-specific determinant in the regulation of Eomes and T-bet cytoplasmic/nuclear compartmentalization. Furthermore, whilst Eomes was reported to partially restore dysfunctional NK cell activity (Gill et al., 2012) by a weak suppression of the inhibitory receptor PD1 in T-cells (McLane et al., 2021), a persistently high Eomes expression has also been associated with increased expression of inhibitory receptor TIGIT in NK cells (Yu et al., 2021). Hence, understanding the pathophysiological triggers for nuclear localization of Eomes will help to conceptualize NK cell function versus exhaustion, for therapeutic purposes. In this study, we used non-small cell lung cancer (NSCLC) cells to delineate the cancer context-specific trigger for Eomes and/or T-bet nuclear localization, and the consequential outcome on NK functions.

The tumour microenvironment (TME) is hypertonic due to high levels of inflammatory cytokines and cations (Burgdorf et al., 2020). The osmolarity of the extracellular compartment has traditionally been known to affect intracellular contractility, with the hypertonic environment triggering increased contractility and vice versa (Guan et al., 2007; Malek et al., 2007). For example, hypertonicity can trigger RhoA-dependent actin reassembly that generates contractile force in endothelial cells (Malek et al., 2007). However, the functional consequences of hypertonicity of the TME on the intracellular contractility of NK cell for mechanosignaling is unknown, especially since the NK cells are non-adherent circulating cells which lack force transduction focal adhesion complexes. Furthermore, mechanotransduction events triggered by intracellular force are able to direct protein structure, function and localization, such as the case of force-induced talin activation at the focal adhesion complex in adherent cells (Lombardi et al., 2011; Carisey et al., 2013; Yao et al., 2016; Elosegui-Artola et al., 2017). However, there is no evidence on whether the cytoplasmic/nuclear localization of transcription factors can be directly influenced by the perturbation of cellular force in non-adherent circulating immune cells like NK when undergoing anti-cancer activities. The activation of NK cells requires actin retrograde flow (Matalon et al., 2018; Santoni et al., 2021) and the convergence and activation of various contractility-promoting proteins such as myosin IIA (Krzewski et al., 2006). Moreover, degranulation of NK cells requires Arp2/3-mediated cytoskeleton rearrangement (Carisey et al., 2018; Santoni et al., 2021). These observations hint that cellular contractility, which awaits exploration, may play a role in regulating protein localization in the activation of NK cells, especially since NK cells harbour abundant cytoplasmic content (Carpen et al., 1982).

NK cells express the TGFβ receptor 1 (TGFβR1) which is activated by TGFβ (Viel et al., 2016), and cancer cells are known to generate high levels of TGFβ (Li et al., 2019). Ample evidence suggests that prolonged exposure to TGFβ downmodulates NK cytotoxicity and promotes its exhaustion (Viel et al., 2016; Zaiatz-Bittencourt et al., 2018). Furthermore, high levels of TGFβ has been associated with poor prognosis of non-small cell lung cancer cell (NSCLC) patients (Li et al., 2019). However, a recent study of peripheral blood γδ T cells showed enhancement of T-cell cytotoxicity with short-term addition of TGFβ (Peters et al., 2019). Collectively, these evidence support a more nuanced view; that the functional impact of TGFβ in immune cells may be spatiotemporally regulated, which may be dissected into potentiating and prolonged phases. Given the important roles of actomyosin-associated proteins in promoting NK cytotoxicity at the immune synapse (Krzewski et al., 2006; Carisey et al., 2018; Matalon et al., 2018), we hypothesized that Eomes and/or T-bet nuclear localization may be associated with NK intracellular contractility beyond the immune synapse, potentially triggered by TGFβ, and this might influence NK cytotoxicity against cancer cells.

Using interdisciplinary approaches of biochemical, biophysical and imaging analyses, we first characterized the expression and localization of Eomes and T-bet in healthy donor NK cells that were challenged with NSCLCs. We found a relatively constant expression of Eomes and T-bet proteins, along with a sustained nuclear localization of Eomes (and to a lesser extent, T-bet). Our findings also demonstrated the non-redundant roles of T-bet and Eomes, where Eomes expression and nuclear localization were specifically associated with enhanced NK cytotoxicity against the NSCLCs. However, prolonged exposure to NSCLCs resulted in imbalance of NK activating and inhibitory receptors, which plausibly reduced the cytotoxic capacity to eliminate the lung cancer cells. Secondly, we performed laser ablation experiments in non-adherent NK cells to demonstrate the presence of actomyosin-mediated intracellular contractility even though the NK cells lack force transduction focal adhesion complexes. We showed that Eomes nuclear localization is driven by enhanced NK intracellular contractility. This is demonstrated by increased F-actin intensity and actomyosin-mediated tensional force, as indicated by a higher F-actin recoil velocity induced by laser-ablation and increased myosin light chain phosphorylation. These observations along with NK cell nuclear flattening, are indicative of increased nuclear pore permeability and higher permissibility for Eomes nuclear localization. Pharmacological perturbation of NK intracellular contractility further confirmed Eomes as the first transcription factor identified to directly respond to cellular forces, resulting in its nuclear localization in NK cells. Thirdly and unexpectedly, we revealed that NK-cancer cell interaction triggered a new disparate role of TGFβ; instead of being immunosuppressive to NK cells, TGFβ sustained an increase in myosin light chain phosphorylation in NK cells, resulting in NK cell contractility which mediated Eomes nuclear localization leading to the enhancement of NK cytotoxicity.

## Results

### NSCLCs Were Susceptible to NK Cell Killing

**FIGURE 1:**
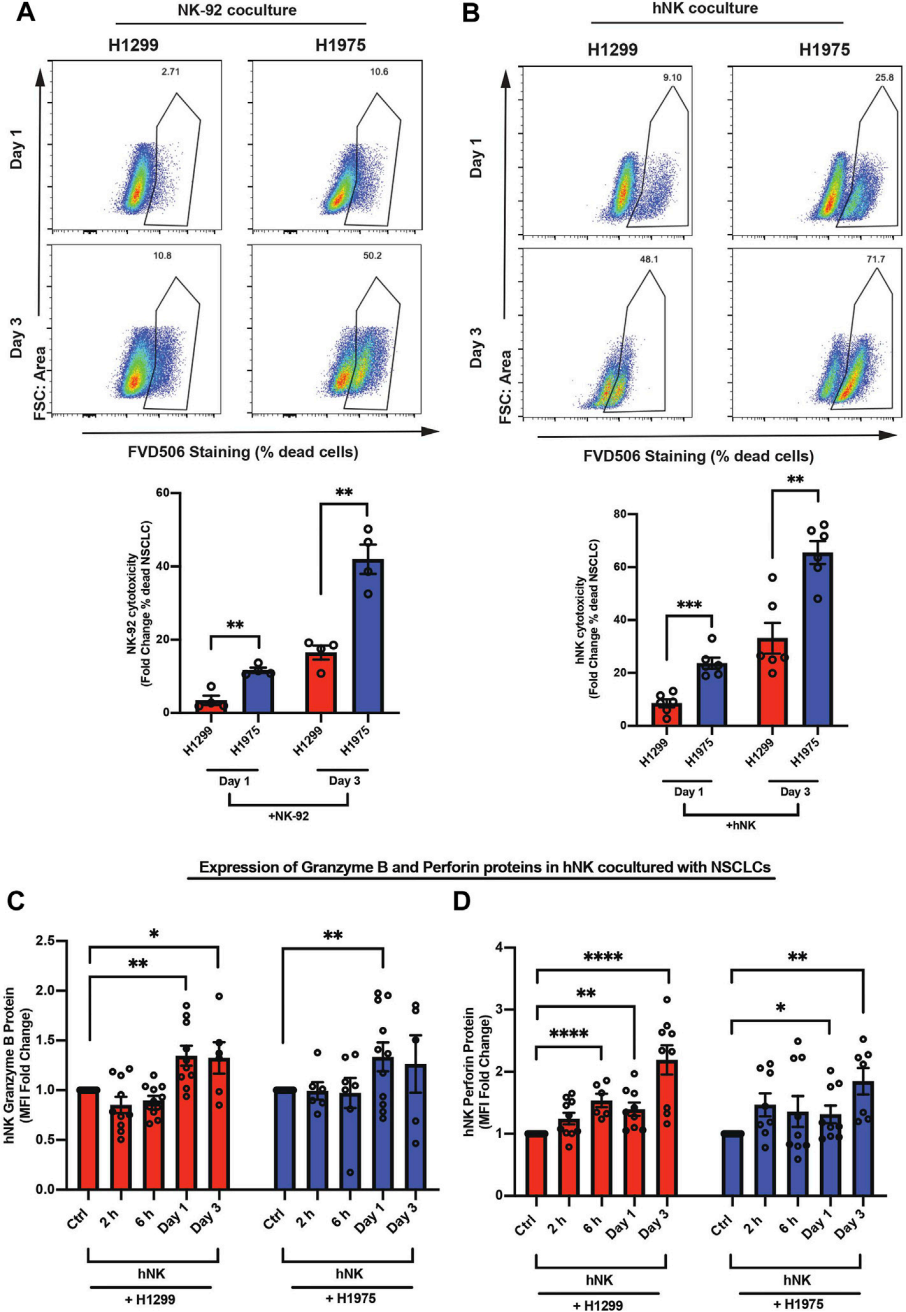
NSCLCs were susceptible to NK cell killing. Flow cytometry analyses show: **(A,B)** Representative and quantified percentage of dead NSCLCs (FVD506 positive). The NSCLCs were CFSE stained prior to coculture with NK-92 **(A)** or hNK **(B)** cells; *n* = 4 for NK-92 coculture. *n* = 6 for hNK coculture. Data are representative of three donors for hNK cells; Gating strategy is presented in Supplementary Figure 1C. **(C,D)** The hNK median fluorescence intensity (MFI) fold change of granzyme B and perforin proteins in hNK cells cocultured with NSCLCs were analyzed by flow cytometry at indicated time points and compared to control hNK cells. The graphs represent means ± SEM; n ≥ 6 and representative of four donors. One way ANOVA test was used to compare across conditions.

The transcription factors, Eomes and T-bet, share a consensus DNA binding T-box domain, but other parts of their sequences are unique (Zhang et al., 2018). To study the mechanistic roles of Eomes and T-bet in NK cells during cancer surveillance, we tested: 1) primary human NK (hNK) cells, which are CD3^−^CD56^+^Tbet^+^Eomes^+^ (Supplementary Figure S1A), and 2) NK-92 cell line, which is Tbet^+^Eomes^+^ (Supplementary Figure S1B), on their anti-cancer capacity against two non-small cell lung cancer cell (NSCLC) subtypes, H1299 (aggressive invasive subtype) and H1975 (less invasive subtype) cells. A coculture setting of cell-cell ratio of 2.5:1 as determined previously (Verma et al., 2020) was established for NK and lung cancer cells, respectively. We found differential killing capacity of hNK and NK-92 against H1299 and H1975 NSCLCs, where H1975 was more susceptible to NK killing (Figures 1A,B). To exclude the possibility that the differential killing capacities of NK cells was due to different proliferation rates of the two NSCLC subtypes, we verified the proliferation marker, Ki67, on NSCLCs cocultured with NK cell. Interestingly, both NSCLC subtypes showed a significant and comparable drop in Ki67 expression in coculture conditions (Supplementary Figures S2A,B). We further verified the invasiveness of the two NSCLC subtypes by comparing their migratory displacements and colony-formation abilities in soft agar. H1299 was significantly more invasive than H1975 (Supplementary Figures S2C,D), which is consistent with a previous report (Shen et al., 2018). Interestingly, at days 1 and 3 of coculture the more invasive H1299 NSCLC induced hNK to produce significantly higher fold-increase in two key targets of Eomes (Araki et al., 2008), perforin (≈2.5 fold) and granzyme b (≈1.5 fold), compared to control hNK cells which were not challenged with NSCLCs (Figures 1C,D). These observations suggest that NK cytotoxicity was upregulated over 1–3 days during which NK cells attempted to kill the NSCLCs, and that there may be an increase in Eomes nuclear localization, resulting in the higher production of perforin and granzyme b. Henceforth, we focused on: 1) the impact of aggressive invasive metastatic NSCLC on NK T-box transcription factors and cytotoxicity, and 2) intrinsic mechanistic characteristics of NK cells during their interaction with the two NSCLC subtypes.

### NSCLC Induced Nuclear Localization of Eomes Within 1 day in NK Cells

We had previously demonstrated that Eomes-low Group 1 ILCs, which contain both NK and ILC1 cell subtypes, are associated with poorer cancer surveillance and worse clinical outcomes for lung cancer patients (Verma et al., 2020). Hence, Eomes probably plays an essential role in the regulation of NK anti-cancer cytotoxicity. Since murine models showed differential expressions of Eomes and T-bet in Group 1 ILCs located in the TME or periphery (Verma et al., 2020), we first analyzed the protein expression of Eomes and T-bet in hNK cocultured *ex vivo* with the NSCLCs. Interestingly, coculture with NSCLCs induced a slight reduction in Eomes expression in hNK and this only occurred after 3 days of coculture with H1299 cells (Supplementary Figures S3A,E). On the other hand, T-bet showed a slight increase at day 3 of coculture with H1975 (Supplementary Figure S3B,F). Nevertheless, these results suggested that any increase in Eomes nuclear localization would not be due to an increase in Eomes protein expression. This is also relevant to translational applications since KIR educating/licensing does not affect T-box protein expression in NK cells (Pradier et al., 2016), and would most likely not affect their expression in NK cells challenged by cancer cells that might experience KIR mismatch issues.

**FIGURE 2:**
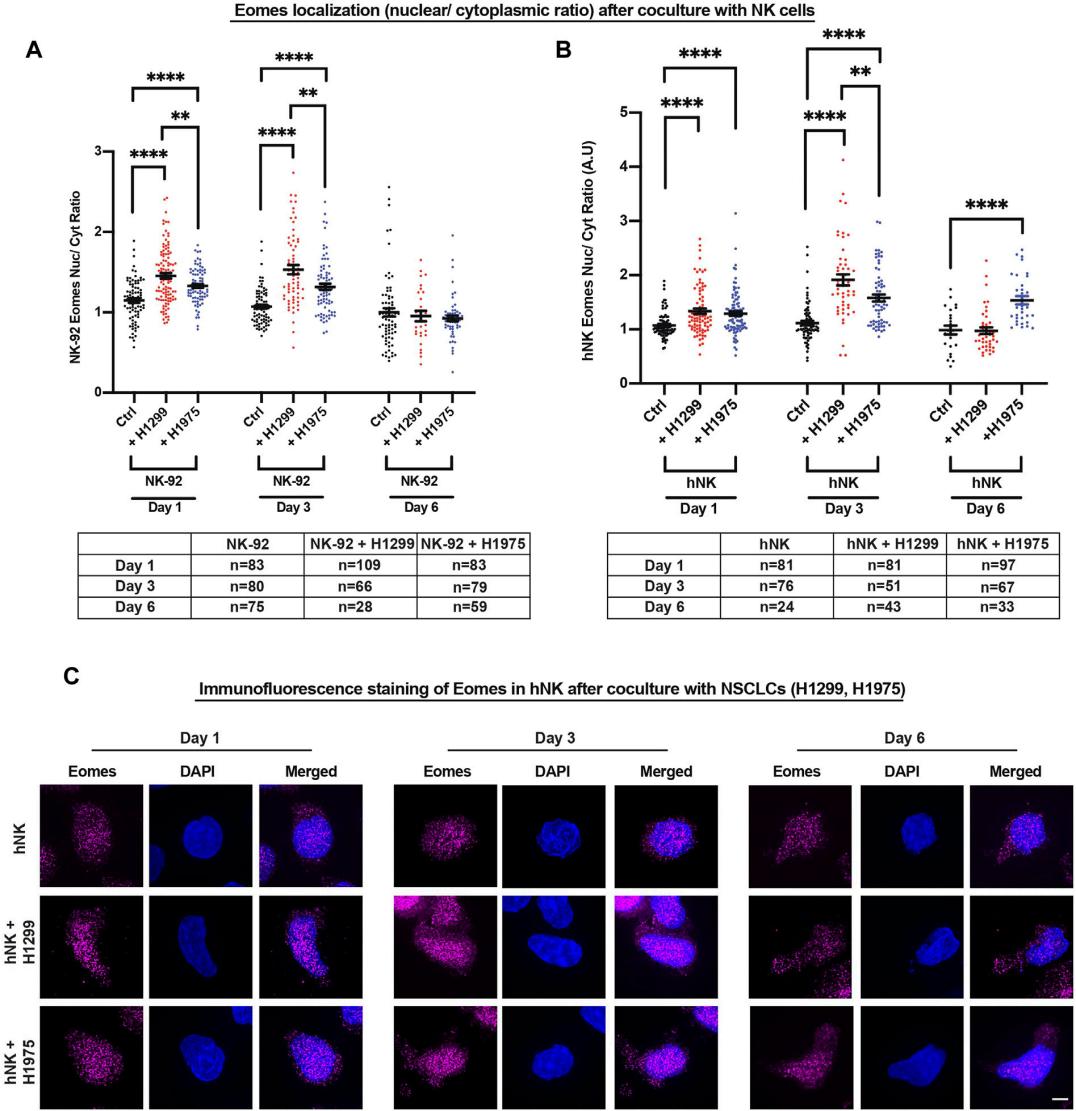
NSCLCs induced nuclear localization of Eomes within 1 day in NK cells**. (A,B)** NK-92 and hNK cells cocultured with NSCLCs showed preferential nuclear localization of Eomes, particularly strongly on day 3 for NK cells cocultured with H1299 NSCLC. The graphs represent means ± SEM; *n* is tabulated below the graphs. For hNK cells, n values are representative of individual cells representative of three donors. One way ANOVA was used to compare the means of each group. **(C)** Representative SIM images of Eomes in hNK cocultured with NSCLCs. Increasing nuclear (DAPI stained) Eomes signal (magenta) is detected in hNK cocultured with NSCLCs. Scale bar = 5 μm.

Since the localization of T-bet and Eomes was previously reported to change in CD8 cytotoxic T cells depending on their activation status (McLane et al., 2013, 2021), we next used a spinning disk microscope equipped with a super-resolution module (Live-SR) to delineate the subcellular localization of Eomes in comparison to T-bet, in three NK cell types (hNK, NK-92 and KHYG-1) cocultured with the NSCLCs. Since there was only a marginal increase in hNK perforin and granzyme b after 2 and 6 h of coculture with NSCLCs (Figures 1C,D), we reasoned that Eomes and T-bet localization would not likely display localization differences at such time-points. Indeed, Eomes or T-bet did not show significant increase in nuclear signals (Supplementary Figure S4A,B). Next, we focused on days 1, 3 and 6 because of significantly higher perforin and granzyme b production observable by day 1 of coculture (monitored from 2 h to 3 days, Figures 1C,D). Consistently, we observed a more prominent nuclear localization of Eomes across all NK cell types (Figures 2 and Supplementary Figure S5) up to 3 days of coculture**.** Interestingly, only T-bet in hNK cells displayed nuclear localization at day 3. However, the nuclear-cytoplasmic ratio of T-bet (≈1.25) (Supplementary Figure S5B **)** was still lower than that of Eomes (≈2.0). Although both NSCLC subtypes induced Eomes nuclear localization in NK cells to different extents, we observed a particularly prominent nuclear localization when hNK cells were primed by the highly invasive metastatic NSCLC, H1299 (Figure 2), corresponding to up to 2.0-fold change in the production of perforin and granzyme b (Figures 1C,D). These results suggest that Eomes nuclear localization (and to a lesser extent, T-bet), may be an attempt by NK cells to upregulate its cytotoxicity. To corroborate these observations, we next cocultured NK-92 cells with two breast cancer cell lines (MCF7 and MDA-MB-231) that were reported to display different susceptibility to NK cell killing (Absi et al., 2018). As expected, the more metastatic and resistant MDA-MD-231 breast cancer cells were able to induce faster and more prominent Eomes nuclear localization in NK-92 cells (Supplementary Figure S4C).

Since Eomes nuclear localization is associated with potentiating NK cytotoxicity (e.g., increased perforin and granzyme b production, Figures 1C,D), we performed cytotoxicity assay against a model target lymphoblast cell line (K562) using two NK cell lines (NK-92 and KHYG-1) which overexpress either Eomes or T-bet, to corroborate that Eomes specifically promotes NK cytotoxicity. In both NK cell types, only Eomes (but not T-bet) overexpression indeed resulted in significantly higher killing of K562 target cells (Supplementary Figure S6).

NSCLCs are one of the leading causes of cancer mortality (Li et al., 2019). Our observation that Eomes did not further display prominent nuclear localization at day 6 of coculture with the invasive H1299 NSCLC (Figure 2), is consistent with reduced NK cytotoxicity after prolonged encounter of NKs with NSCLCs. One possible explanation is the regulation of surface activating and inhibitory receptors of NK cells, which is well-associated with NK exhaustion and reduced function (Gill et al., 2012; Lee et al., 2021). To verify this possibility, we analyzed the surface expression of various inhibitory (PD-1, TIGIT, NKG2A) and activating (NKG2D) receptors on viable (FVD506-negatively stained) hNK and NK-92 cells cocultured with NSCLCs. Interestingly, there was a consistent reduction in all receptors analyzed, and especially so for hNK cells (Supplementary Figure S7 **).** These results suggest that prolonged exposure to invasive metastatic NSCLCs progressively imbalances NK receptors, rendering Eomes nuclear localization insufficient to overcome invasive cancer cells. Taken together, these data suggest a specific role of Eomes, in activating a dormant cytotoxic machinery by translocating into NK nucleus. However, such mechanism was undermined in prolonged coculture setting, possibly due to the imbalance in activating and inhibitory receptors. These observations warrant a deeper understanding on the extrinsic and intrinsic signaling mechanism that trigger NK Eomes nuclear localization when they encounter NSCLCs.

### NSCLCs Activated NK Cellular Contractility and Induced Nuclear Flattening

**FIGURE 3:**
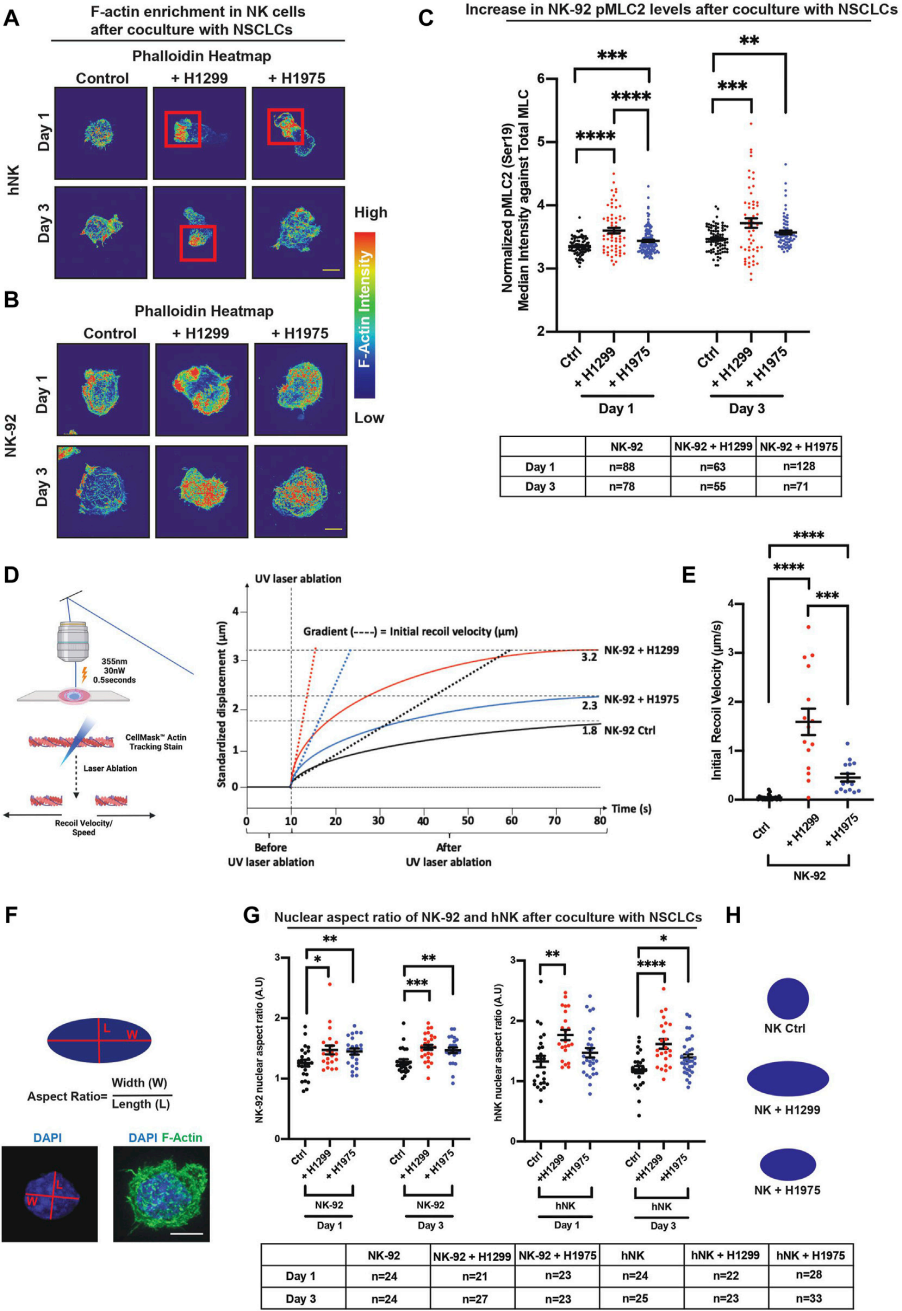
NSCLCs activated NK cellular contractility and induced nuclear flattening. **(A)** SIM super-resolution images show localised enhancement of F-actin concentration in hNK cells that were cocultured with NSCLCs. Red squares highlight areas of F-actin enrichment in hNK cells that were cocultured with NSCLCs. All samples were stained with a master mix of phalloidin, specific for F-actin, and laser power and exposure settings were kept constant across samples imaged. Scale bar = 5 μm. **(B)** NK-92 cells showed an increase in F-actin intensity after coculture with NSCLC, corresponding to an increase in F-actin heatmap on the right. All samples were stained with a master mix of phalloidin and laser power and exposure settings were kept constant across samples imaged. Scale bar = 5 μm. **(C)** Cocultures of NK-92 cells with NSCLCs increased phosphorylation of myosin light chain 2 at Ser19 (pMLC2) in NK-92 cells. All samples were stained with a master mix of respective primary and secondary antibodies and imaged with same laser power and exposure time. Representative images are shown in Supplementary Figure S8B. Unpaired students *t*-test was used to compare between conditions. The graphs represent means ± SEM and n is tabulated below the graph. **(D)** Schematics showing laser ablation of NK cells, and representative graph showing maximum recoil displacement (Ctrl = 1.8 μm, + H1299 = 3.2 μm, and + H1975 = 2.3 μm) of laser ablated actin, and the derivation of initial recoil velocity. Detailed experimental and quantification procedures are documented in the materials and methods section. **(E)** NK cells cocultured with NSCLCs show higher F-actin recoil velocity compared to control NK cells unchallenged by NSCLCs. Unpaired students *t*-test was used to compare F-actin recoil velocities between conditions. The graphs represent means ± SEM and *n* = 24 for control, and *n* = 15 for NSCLC coculture. **(F)** Schematic showing quantification of aspect ratio as a representative indicator for nuclear flattening. Blue signal represents the nucleus. Scale bar = 5 μm. **(G)** NK-92 and hNK cocultured with NSCLCs consistently showed increased nuclear aspect ratio (nuclear flattening). The graphs represent means ± SEM, n is tabulated below the graph. Unpaired students *t*-test was used. **(H)** Schematic representing nuclear flattening with an increased aspect ratio after NK coculture with NSCLCs. Dark blue schematic represents the nucleus of NK cells.

The biological roles of actomyosin in NK cells has thus far been focused on its regulation of the immune synapse (Krzewski et al., 2006; Carisey et al., 2018; Jankowska et al., 2018). However, information on how global regulation of actomyosin-mediated cellular contractility may affect NK function, is unexplored albeit important. Intracellular actomyosin-based contractility has been proposed to regulate protein activities through focal adhesion complex present in adherent cells (Elosegui-Artola et al., 2017). Yet, such contractility-based regulation of protein activation and localization still remains an undefined physiological trigger. Based on our preliminary findings, we hypothesized that when NK cells encounter NSCLCs, the actin dynamics would impel NK cell contractility, which will in turn impact the subcellular compartmentalization of Eomes. To investigate this possibility, we first cocultured NK cells with the two NSCLC subtypes, H1299 and H1975, for 1 and 3 days. A high Eomes nuclear localization signal and high levels of perforin and granzyme b were observed at these timepoints (Figures 1C,D, Figure 2). We next characterized the F-actin organization in NK cells after coculture. In the absence of NSCLC, the F-actin in the hNK cell was observed to be relatively homogeneous in the cell body with occasional filipodia-like structures (Figure 3A). However, for hNK cocultured with NSCLCs, we observed a notable display of F-actin polarization and condensation (Figure 3A, red box and Supplementary Figure S8A). Interestingly, the invasive metastatic H1299 cells were found to sustain such polarization over 3 days whereas the less invasive H1975 reverted hNK to a control-like phenotype (Figure 3A and Supplementary Figure S8A). Similar observations were made with NK92-NSCLC coculture, where NK92 displayed an increase in F-actin intensity (Figure 3B). With the less invasive H1975, the NK cell displayed a correspondingly lower intensity of actin at day 3, which was also consistent with lower translocation of Eomes to the nucleus (Figure 2). These observations suggest enhancement of intracellular contractility in NK cells upon encountering NSCLCs.

Since high nuclear localization of Eomes is associated with more pronounced condensation of F-actin in NK cells (Figure 2, Figures 3A,B), we next sought to understand the functional association between higher F-actin and intracellular contractility on the localization of Eomes. The Rho-ROCK-Myosin pathway is known to increase cellular contractility, and myosin has been identified to stabilize cellular F-actin (Amano et al., 1996, 2010; Yamashiro et al., 2018). Thus, we analyzed the phosphorylation status of the non-muscle myosin II light chain 2 (MLC2) in NK cells cocultured with NSCLCs to ascertain if there was an increase in cellular contractility. Indeed, despite relatively constant staining of total MLC2 (Supplementary Figure S8B,C), we observed a prominent rise in the normalized expression (intensity) of phosphorylated MLC2 (pMLC2) in NK cells cocultured with NSCLCs (Figure 3C and Supplementary Figure S8B). Consistently, H1299 NSCLC appeared to have primed NK cells to sustain a significantly higher level of pMLC2 for up to 3 days, concurrent with the peak of nuclear Eomes localization (Figure 2A).

To directly determine whether the increase in F-actin intensity and myosin light chain phosphorylation result in enhanced NK intracellular contractility, we analyzed the recoil velocity of F-actin by performing a laser-ablation experiment on NK-92 cells cocultured with NSCLCs (Figure 3D). Corroboratively, we found that NK cells cocultured with NSCLCs displayed profound and significant recoil velocities compared to control NK cells alone, which is a direct indication of a stronger intracellular contractile force generated by the actomyosin complex (Figure 3E). Consistently, we observed a higher recoil velocity in NK cells cocultured with H1299 compared to H1975, and this corresponds to higher F-actin intensity and myosin light chain phosphorylation when NK cells encounter the more aggressive NSCLC.

Since the nucleus is mechanically coupled to the cytoskeleton via the LINC complex (Guilluy et al., 2014), an increase in intracellular contractility will tether mechanical force transmission to the nucleus and result in nuclear flattening with consequential increase in the nuclear aspect ratio (Elosegui-Artola et al., 2017; Alisafaei et al., 2019). Changes in nuclear geometric parameters would favour increase in nuclear pore permeability and protein import (Elosegui-Artola et al., 2017; Alisafaei et al., 2019). Thus, we compared the aspect ratios (length/width) of the NK nucleus, between control NK and NK + NSCLC cocultures (Figure 3F). Interestingly, the more aggressive metastatic H1299 NSLC flattened the NK nucleus starting from day 1, yielding a more prominent aspect ratio (≈40% more than control) by day 3 (Figures 3G,H). On the other hand, hNK cocultured with H1975 showed a moderate nuclear flattening at day 3 (Figures 3G,H), again corresponding to the lesser nuclear Eomes observed at day 3 (Figure 2). Similar observations were made with NK-92 cocultures (Figures 3G,H). Altogether, these data suggest that compounding factors in the coculture media could have resulted in a graded response within NK cells that differentiates between invasive and less invasive cancer cell lines. For instance, the fine tuning of cellular contractility through myosin light chain phosphorylation could be one of the mechanisms involved. Nevertheless, nuclear flattening observed in NK cells suggested that this is associated with mechanical deformation of NK nucleus which favours shuttling of Eomes into the nucleus.

### Eomes Is the First Transcription Factor Identified to Respond to Cellular Contractility

**FIGURE 4:**
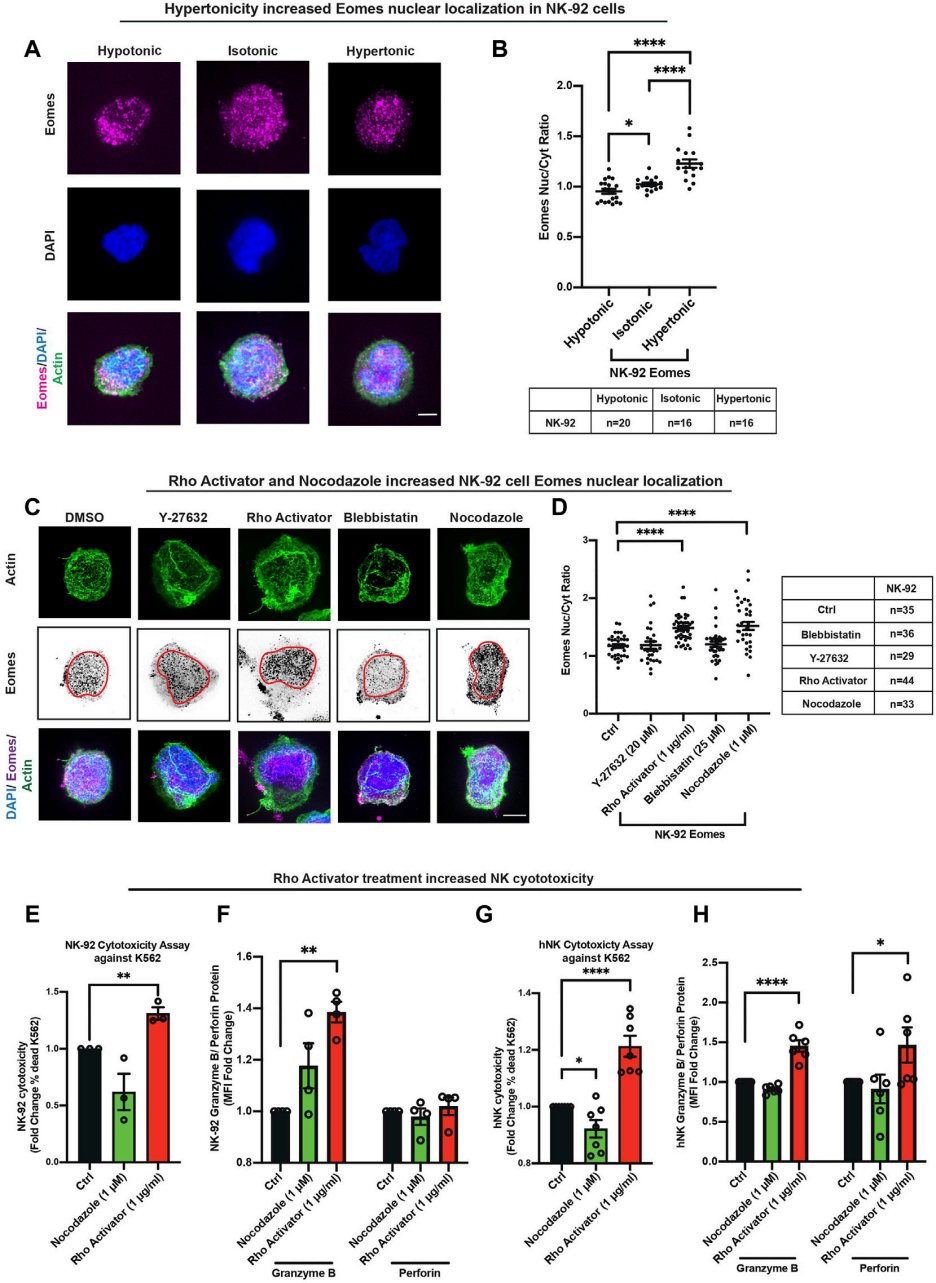
Eomes is the first transcription factor identified to respond to cellular contractility**. (A,B)** Hypertonic treatment significantly increased NK-92 Eomes nuclear localization. Representative images and quantification of Eomes intracellular localisation after 30 min of tonicity treatment. The graph represents means ± SEM, *n* is tabulated below the graph. Scale bar = 5 μm. Unpaired students *t*-test was used. **(C,D)** Representative images and quantification on the right show increased nuclear localisation of Eomes with Rho activator and nocodazole treatment. The graph represents means ± SEM, *n* is tabulated beside the graph. Scale bar = 5 μm. Unpaired students *t*-test was used. **(E,G)** Increased cellular contractility due to treatment with Rho activator enhanced NK-92 and hNK cytotoxicity (reflected as percentage killing) against model target K562 cells, effector (NK): target (K562) ratio was 3:1 for NK-92 and 1:1 for hNK cells. *n* = 3 for NK-92 and *n* = 7 for hNK, representative of three donors. **(F,H)** Rho activator increased the production of granzyme B in NK-92 **(F)** and hNK **(H)** cells cocultured with K562 cells, whereas only hNK cells showed an increase in perforin expression with Rho activator treatment. *n* = 4 for NK-92 cells and *n* = 6 independent experiments representative of three donors for hNK cells. All graphs are represented by means ± SEM and unpaired students *t*-test was used.

Because enhanced NK intracellular contractility is associated with increased Eomes nuclear localization, we reasoned that intracellular contractility alone might be sufficient to drive Eomes nuclear shuttling. To test this hypothesis, we subjected NK-92 cells to iso-, hypo-, or hyper-osmotic shocks and stained for actin with phalloidin (Supplementary Figure S9A). Since the *in vivo* TME (represented partially in these experiments by the conditioned coculture medium) is hypertonic due to chronic inflammation which supports an intratumoral osmotic pressure (Sirtl et al., 2018; Burgdorf et al., 2020), there is a direct physiological link to the role of increased NK cell contractility in promoting Eomes nuclear localization, which plausibly regulates NK cytotoxicity. Thus, we employed a hypertonic treatment, which will induce a flaccid membrane with a concurrent increase in intracellular contractility, and vice versa with hypotonic treatment (Gonano et al., 2014). Interestingly again, only Eomes (and not T-bet) specifically responded to hypertonicity associated with increased nuclear localization observed (Figures 4A,B). In fact, T-bet only displayed moderate localization differences between extreme osmolarities, viz, hypotonic and hypertonic conditions (Supplementary Figures 9B,C).

The phosphorylation of the myosin light chain directly contributes to actomyosin-based intracellular contractility (Ridley, 2001), which is conceivably associated with Eomes nuclear localization. Thus, we performed pharmacological perturbation of this pathway to ascertain Eomes (using T-bet as control) nuclear localization in response to altered intracellular contractility. We treated NK-92 cells with contractility perturbation drugs, Y-27632 (ROCK inhibitor) and Rho Activator, targeting the Rho-ROCK-Myosin pathway, and quantified Eomes and T-bet nuclear localization. Although Rho activation could have induced several molecular pathways (Bros et al., 2019; Wurzer et al., 2019), its constitutive activation was shown to induce abundant stress fiber and enhance intracellular contractility (Hodge and Ridley, 2016). As expected, treatment with Y-27632 marginally reduced the nuclear localization of Eomes (Figures 4C,D) and T-bet (Supplementary Figures S9D,E). Notably, Rho activator, the upstream small GTPase that activates myosin through ROCK kinase, significantly increased Eomes nuclear localization (Figures 4C,D). To corroborate this, we verified NK-92 Eomes localization by treating the cells with blebbistatin and nocodazole. Blebbistatin inhibits myosin activity and nocodazole induces the release of the Rho GTPase exchange factor, GEF-H1, which increases intracellular contractility (Pan et al., 2020). Indeed, there was a significant increase in Eomes nuclear localization with nocodazole treatment (Figures 4C,D).

Since increased intracellular contractility specifically impels Eomes into the nucleus, and upregulates NK cytotoxicity (Supplementary Figure S6), we hypothesized that an increase in contractility would functionally upregulate NK cytotoxicity. Indeed, cytotoxicity assay of NK cells pre-treated with Rho activator was shown to significantly increase NK cytotoxicity against the target K562 lymphoblast cell line, and this was associated with increased production of the Eomes transcription target, granzyme B (Figures 4F,H). Furthermore, there was a consistent reduction in the surface expression of the inhibitory receptors TIGIT and NKG2A (Supplementary Figure S9F). Although nocodazole pre-treatment increases contractility and Eomes nuclear localization, it is a potent microtubule depolymerization reagent that could have affected multiple cellular processes. This explains a reduction in NK cytotoxicity with nocodazole pre-treatment (Figures 4E,G).

Taken together, we demonstrate for the first time that a transcription factor, Eomes, responds directly to intracellular contractility perturbations via the Rho-ROCK-Myosin pathway. Although the NK cells are a non-adherent circulating cell type, which lack the force transduction focal adhesion, their ability to summon intracellular contractility for Eomes to gain traction into the nucleus, is unique and interesting in view of NK cell reactivation. This prompted us to further identify the extrinsic and/or intrinsic trigger(s) of intracellular contractility-mediated Eomes nuclear localization induced in *ex vivo* NK-NSCLC coculture, with a view to elucidating the mechanisms underlying such a promotion of NK cytotoxicity against the invasive NSCLC subtype.

### TGFβ Induced NK Cell Contractility and Eomes Nuclear Localization

Metastatic NSCLCs are known to be associated with high levels of the presumably immunosuppressive cytokine TGFβ (Ye et al., 2015). Nonetheless, observations in non-immune systems suggest the induction and activation of contractile genes (Birukova et al., 2005; Ojiaku et al., 2018; Gladilin et al., 2019), which has yet to be proven in immune cells. This prompted us to examine a potential opposing/disparate role of TGFβ during the early phase of NK anti-cancer coculture, which may facilitate the activation of NK cytotoxicity.

**FIGURE 5:**
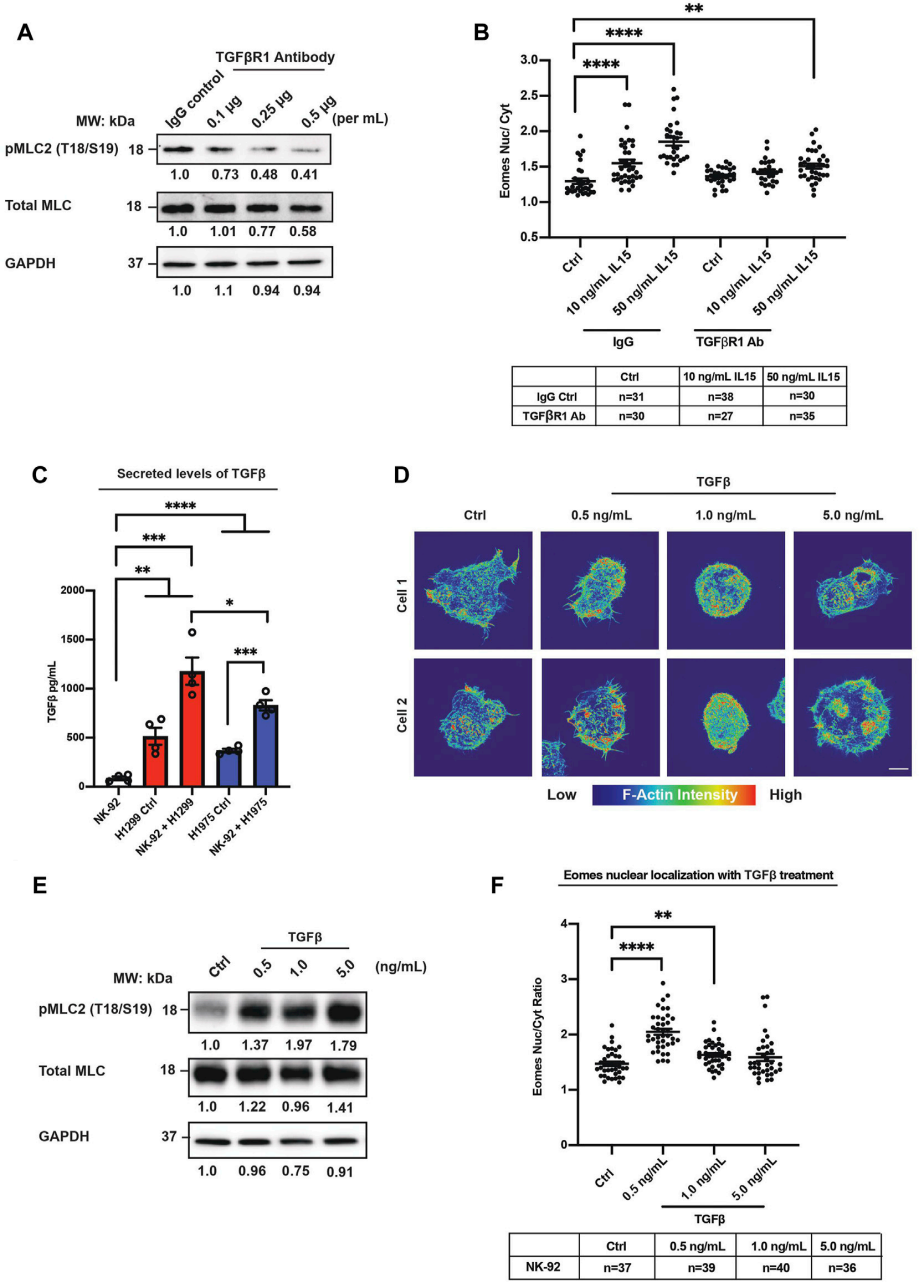
TGFβ-induced myosin light chain phosphorylation is associated with Eomes nuclear localization**. (A)** Western blot with densitometric quantification (numerically represented below each band) shows that TGFβ1R antibody blocking reduced pMLC2 (Thr18/Ser19) expression in a dose-dependent manner, *n* = 3. **(B)** IL15 induced dose-dependent increase in Eomes nuclear localization in NK-92 cells, whereas TGFβR1 antibody blocking (compared to control IgG) suppressed IL15-induced nuclear localization of Eomes in NK-92 cells. n is represented in the table below the graph. One way ANOVA was used to compare the means of each group. **(C)** ELISA quantification of TGFβ in supernatants from individual cultures and cocultures showed higher TGFβ levels in the presence of H1299 cells, *n* = 4. Unpaired students *t*-test was used to compare between groups. **(D)** SIM super-resolution images show enhancement of F-actin concentration in NK-92 cells that were pre-treated with TGFβ. The scale below represents the color coding for actin intensity. All samples were stained with a master mix of phalloidin, specific for F-actin, and laser power and exposure settings were kept constant across samples imaged. Scale bar = 5 μm. **(E)** Western blot with densitometric quantification (numerically represented below each band) shows TGFβ increased pMLC2 (Thr18/Ser19) expression in a dose-dependent manner, *n* = 3. **(F)** TGFβ treatment promoted nuclear localization of Eomes in NK-92 cells in a dose-dependent manner as indicated by higher Eomes nuc/cyt ratio. n is tabulated below the graph. All graphs are represented by means ± SEM and unpaired students *t*-test was used.

Since coculturing with NSCLC induced myosin light chain phosphorylation in NK cells (Figure 3C), we first verified if blocking the TGFβR1 receptor will affect MLC2 phosphorylation in NK cells. Indeed, treatment with anti-TGFβ1R blocking antibody exhibited a dose-dependent reduction of pMLC2 in NK-92 cells (Figure 5A), suggesting that TGFβR1 activation is responsible for maintaining NK contractility through myosin light chain phosphorylation. Importantly, we showed that blocking TGFβR1 with a high dose of anti-TGFβR1 antibody did not affect Eomes and T-bet expression levels (Supplementary Figure S10A **)**, implying that any observed regulation of Eomes and/or T-bet localization through TGFBR signaling is not attributed to changes in their protein levels. Next, we directly verified if blocking TGFβ1R on NK cells would affect Eomes nuclear localization (using T-bet as control). Consistent with our hypothesis, we demonstrated that inhibition of TGFβ1R specifically reduced Eomes nuclear localization in NK-92 cells stimulated with PMA/Ionomycin, conceivably attributable to reduced cellular contractility (Supplementary Figure S10B), whereas again, there was no change in T-bet nuclear localization (Supplementary Figure S10C). To corroborate the finding that TGFβ1R blocking could affect Eomes nuclear localization, we stimulated NK-92 cells with a physiological stimulant, IL15. IL15 was previously shown to synergize TGFβ activities in immune cells (Hawke et al., 2020). We found that treatment of NK-92 with IL15 induced a dose-dependent increase in Eomes nuclear localization. However, such increase was less prominent when NK cells were pre-treated with TGFβ1R blocking antibody (Figure 5B). These results suggest that there could be an increased TGFβ production and/or TGFβ1R surface expression in coculture conditions which might have facilitated the increase in cellular contractility in NK cells.

The above findings prompted us to analyze the mRNA expressions of *TGFB1* and *TGFBR1* genes from the flow sorted NK-92 and NSCLCs after coculture (Supplementary Figures S10D,F). Consistently, H1299 cells expressed a higher level of *TGFB1* mRNA compared to H1975 cells in both control and coculture conditions (Supplementary Figure S10F). Interestingly, H1299 cells induced >10-fold increase in *TGFBR1* mRNA in NK-92 cells (Supplementary Figure S10D) compared to that induced by H1975. These results suggest that the innately high level of TGFβ1 produced by the invasive metastatic NSCLC (H1299) markedly induced the expression of NK cell TGFβR1, to further enhance NK cell contractility through myosin light chain phosphorylation. To corroborate these observations, we further verified surface level of TGFβR1, which indeed showed a higher expression on NK-92 cells that were cocultured with the invasive metastatic H1299 NSCLC compared to that of coculture with H1975 NSCLC (Supplementary Figure 10E).

Next, we performed an ELISA to determine the secreted levels of TGFβ in NK-92, H1299 and H1975 alone cultures and in coculture conditions. As shown in Figure 5C, both cocultures with H1299 and H1975 significantly increased the secreted levels of TGFβ1, with the more invasive H1299 inducing a substantially higher level of secreted TGFβ1. Interestingly, the secreted levels of TGFβ in NK-92 or NSCLC alone media did not add up to that of the coculture conditions (Figure 5C). Furthermore, there was an increase in NK-92 *TGFB1 mRNA in* coculture conditions (Supplementary Figure 10D). These observations suggest that the source of TGFβ could be contributed by both NK and NSCLCs and we cannot rule out autocrine regulation of NK cells by its TGFβ secreted into the media. However, this warrants full scale studies in future investigations.

Since the enhanced contractility and Eomes nuclear localization may be in part due to TGFβ1 in the culture media, we reasoned that the TGFβ1 level determined by ELISA (Figure 5C) should be able to stimulate actin reorganization, myosin light chain phosphorylation and induce significant Eomes nuclear localization. We first asked whether actin reorganization and density would be affected. We found that treatment with a low dose (0.5 ng/ml) of TGFβ1 was sufficient to induce significant increase in actin reorganization and filopodia-like protrusions from NK-92 cells (Figure 5D), which was indicative of RhoA activation (Heasman and Ridley, 2010). Furthermore, actin reorganization after treatment with TGFβ1 was also associated with a prominent increase in myosin light chain phosphorylation (Figure 5E). Finally, we verified that Eomes displayed highest nuclear localization at the doses of 0.5 ng/ml and 1.0 ng/ml (Figure 5F), corresponding to TGFβ levels in the culture supernatants of H1299 alone and coculture with H1299, respectively (Figure 5C).

**FIGURE 6:**
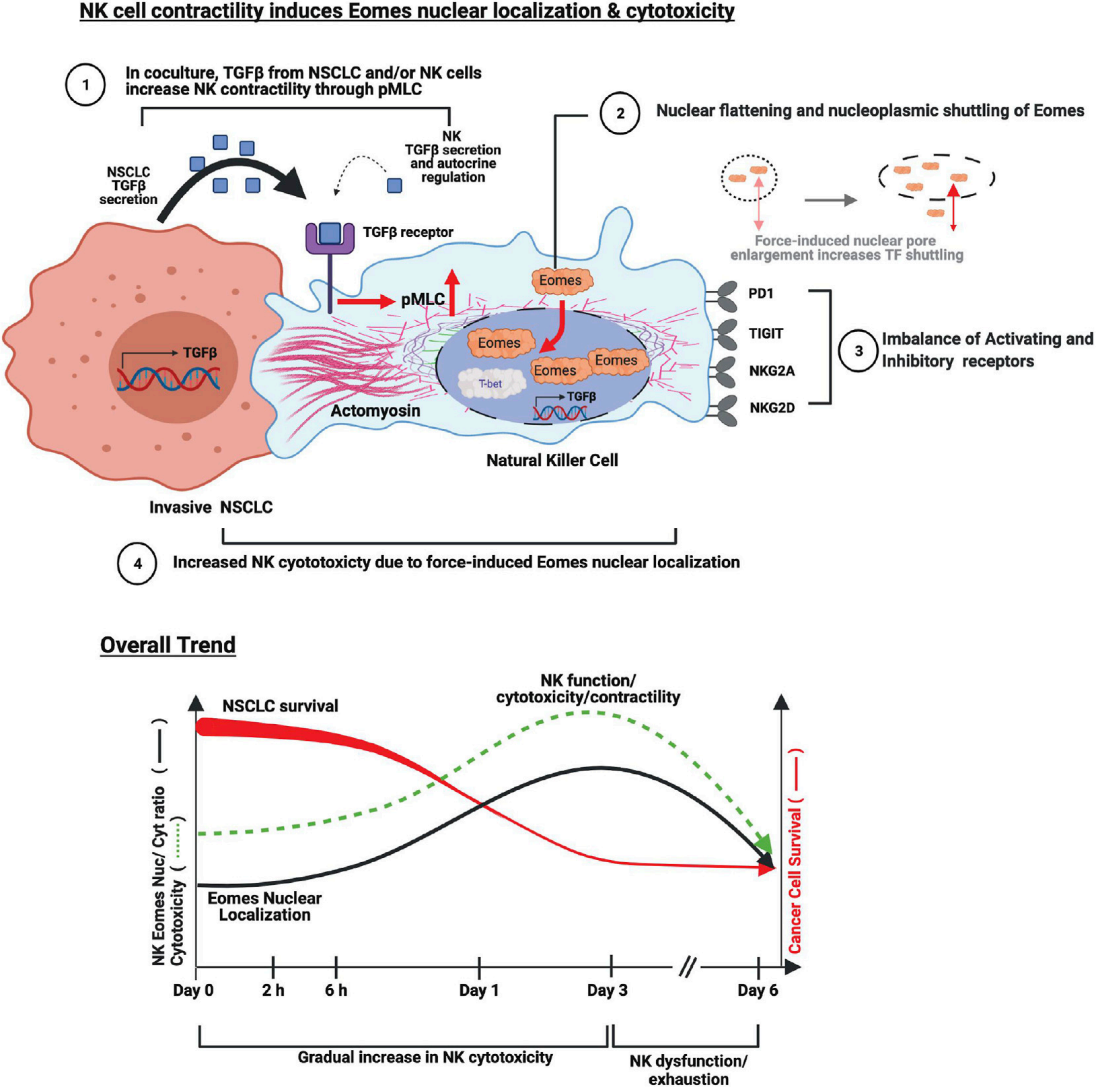
A model summarizing NK cellular contractility which induces Eomes nuclear localization and provokes antitumor surveillance. (Cell-molecular mechanism) Metastatic invasive NSCLC (e.g., H1299) produces higher levels of TGFβ to which NK cells respond by increasing TGFβR1 expression, which activates downstream signal transduction events. (1) Continuous production and secretion of TGFβ persistently increases NK cell contractility through TGFβR1 upregulation and MLC phosphorylation (pMLC). The source of TGFβ could be from NSCLCs and/or NK cells, the latter of which could conceivably mediate autocrine regulation of NK cell activity, as evident by an increase in TGFβ mRNA; (2) Enhanced cellular contractility (as indicated by actin-recoil) promotes actin re-organization and Eomes nuclear shuttling. One mechanism is through mechanical stretching of the nuclear pore, which promotes transcription factor (TF) shuttling (Elosegui-Artola et al., 2017); (3 and 4) Imbalance of activating and inhibitory receptors on NK cell surface reduced the cytotoxicity of NK cells despite increased Eomes nuclear localization, which compromises NK cell killing of metastatic NSCLC. The insufficiency/exhaustion of NK cells is exacerbated by continuous TGFβ secretion by the resistant invasive NSCLC. (Overall Trend) NSCLCs increase NK contractility which is accompanied by nuclear shuttling of Eomes that surged from day 1. The increase in NK cytotoxicity is sufficient to subdue the less invasive H1975 NSCLC and partially eliminate the more invasive H1299. However, as Eomes nuclear residency peaked at day 3, NK cytotoxicity against the more invasive metastatic H1299 wanes, possibly due to compounding factors present in the highly immunosuppressive microenvironment, eventually leading to exhaustion-like phenotype.

To recapitulate, we have shown that in the presence of NSCLCs, the NK cells increased their cellular contractility which potentiated Eomes nuclear localization associated with enhanced cytolytic activity against the NSCLCs. Mechanistically, Eomes responds to actomyosin-mediated contractility, and preferentially localizes to the nucleus of NK cells. The sustained NK cell contractility is explained by the trigger from secreted TGFβ (ligand) from NK and NSCLCs, and NK cell TGFβ1R (receptor) which evoked pMLC2-mediated NK cell contractility to increase Eomes nuclear localization. Overall, the basal cytotoxicity of NK cell with moderate Eomes nuclear localization was only sufficient to subdue the less invasive NSCLC (e.g., H1975). It is plausible that NK cells exploit and contravene the seemingly immunosuppressive tumor microenvironment (represented here as conditioned culture medium from NK + NSCLC coculture) to potentiate its cytolytic response over 1–3 days, but this diminished thereafter due to the imbalance in NK activating and inhibitory receptors. Figure 6 illustrates our findings in a schematic model.

## Discussion

In our previous study, we found that Eomes-low Group 1 ILCs in mice, which contain both NK and ILC1 cell subtypes, are associated with poorer lung cancer surveillance and worse clinical outcomes (Verma et al., 2020). However, recent evidence shows that human NK cells in the TME display similar Eomes and T-bet expression compared to those in the periphery (Russick et al., 2020). Hence, by using hNK cells from healthy donors, we characterized differential intracellular localization of these transcription factors that exist in the context of anti-NSCLC activities and provide a mechanistic insight on the extrinsic roles of TGFβ in the regulation of NK actomyosin contractility during this process. We discovered that compared to the less invasive H1975, the more invasive H1299 NSCLC could increase and sustain Eomes nuclear localization, which was associated with increased NK cytotoxicity (indicated by granzyme b and perforin production) against NSCLCs. Furthermore, when in the presence of H1299, higher levels of TGFβ triggered phosphorylation of myosin light chain 2 (pMLC2), resulting in an increase in F-actin condensation, which upregulated NK intracellular contractility for nuclear localization of Eomes. However, prolonged exposure to uncleared NSCLC ultimately led to an imbalance in cell surface activating and inhibitory receptors on the NK cells. Collectively, we propose an alternative view - that at the appropriate and subtle concentration, TGFβ spatiotemporally acts as an NK cell activator through the activation of actomyosin-mediated contractility for Eomes nuclear localization. Additionally, we propose Eomes in NK cells to be the first mechanosensitive transcription factor identified to regulate anti-cancer activities.

In agreement with an earlier study (Chang et al., 2011) which reported that T-bet was predominantly localized to the T-cell nucleus, we observed only little compartment shuttling of T-bet in NK cells undergoing antitumor activities. However, in contrast to a study on human CD8 T-cells (McLane et al., 2013), we demonstrated a key and novel mechanistic finding, that with human NK cells, Eomes specifically displays nuclear compartmentalization preference when challenged by NSCLCs and this was not due to an increase in Eomes protein expression. Intriguingly, despite an increase in the level of nuclear Eomes, the NK cells were somewhat constrained in eliminating the more invasive NSCLC H1299. This seemed to undermine our initial finding that overexpression of Eomes promoted cytotoxicity against the lymphoblast model target cell line, K562. It also seemed counter-intuitive of the expectation that Eomes overexpression in adoptively transferred murine NK cells increases cytotoxicity (Gill et al., 2012). On the other hand, we have also shown that Eomes nuclear localization is associated with reduction of PD-1 expression, agreeing partially with the previous finding that Eomes and T-bet are suppressors of the *PDCD1* gene in CD8 T-cells (McLane et al., 2021).

Although it was reported that Eomes sustained or enhanced the expression of several inhibitory receptors and promoted NK (Gill et al., 2012) and T cell (Kurtulus et al., 2015) exhaustion, these experiments typically entail long durations (e.g., 17 days). Furthermore, the effects observed were attributed to Eomes overexpression or downregulation. We have shown in our studies that Eomes expression in NK cells remained relatively constant throughout the coculture period. Hence, any perceived change (generally decreasing trend) to receptor expressions could be due to direct or indirect consequences of the subcellular localization of Eomes (nuclear/cytoplasmic translocation). Interestingly, it was reported that PD-1 blockade in CD8 T-cells induced higher expression of Eomes related genes, and this process was proposed to be a consequence of increased Eomes nuclear localization after PD-1 blockade (McLane et al., 2021), which corroborates our inference. Although progressive exhaustion cannot be ruled out as a possible mechanism that limited NK cytotoxicity, we did not observe an upregulation of inhibitory receptors, but rather, downregulation of both the inhibitory and activating receptors in prolonged cocultures. Such observations suggest that either: 1) NK cells reduced their cytotoxicity for self-preservation; this prevents over-activation due to persistent reduction in inhibitory receptors and/or 2) the inhibitory and activating receptors might co-regulate the activities of NK cells when overwhelmed by the persistently immunosuppressive condition. Indeed, several NK cell activating and inhibitory receptors were identified to share/compete for the same ligands (Chauvin and Zarour, 2020; Holder and Grant, 2020), suggesting additional fine-tuning of downstream signaling. In addition, actin remodeling in more invasive NSCLCs could also be a limiting factor against NK cytotoxicity. For instance, it was recently reported that metastatic breast cancer cells were enriched in synaptic actin responses that served as a protective barrier against NK cell attack (Absi et al., 2018). Interestingly, we have also shown in our study that Eomes nuclear localization was more prominent in NK cells challenged with the more invasive breast cancer cells, MDA-MB-231 (see Supplementary Figure S4C). Such consistent observations on NK cell response against different cancer types (lung and breast cancers) are interesting and warrant future investigation since synaptic actin responses in target cells are associated with altered NK receptor ligand density (Absi et al., 2018). It is also entirely possible that any increase in TGFβ levels in the coculture could similarly induce activation of cancer cell contractility, which could in turn affect cancer cell ligand density to facilitate the evasion from NK cell killing.

Our findings also highlight two plausible physiological triggers (hypertonicity and TGFβ) and their functional consequences which promoted the contractility-induced nuclear compartmentalization of Eomes. The pathophysiological TME is hypertonic due to chronic inflammation which supports an intratumoral osmotic pressure (Sirtl et al., 2018; Burgdorf et al., 2020). Hypertonicity was earlier demonstrated to increase adherent cell intracellular contractility (Gauthier et al., 2011; Gonano et al., 2014). Here, we provide the first evidence that human circulating immune cells are mechanosensitive and harness contractility as a means to induce nuclear translocation of transcription factors to elicit cytotoxicity against NSCLCs. This may explain why previous studies showed a positive graded response of immune response genes (including *IFNG*) when immune cells (e.g., T-cells) were cultured on substrates of increasing stiffness (Saitakis et al., 2017; Santoni et al., 2021). Also, our findings help explain why an increase in osmolarity due to hemorrhages (Pirkle and Gann, 1976) can result in the activation of infiltrating NK cells that exhibit increased cytotoxicity and chemokine production versus peripheral NK cells (Li et al., 2020).

We further identified a spatiotemporally graded response initiated by the TGFβ, to sustain intracellular contractility by promoting myosin light chain phosphorylation, thereby evoking Eomes nuclear entry. This is supported by decreased Eomes nuclear localization upon blocking of the TGFβR1 receptor and increased Eomes nuclear localization with TGFβ treatment. Our data seem to contradict immune studies showcasing TGFβ as a potent immunosuppressive cytokine, particularly towards NK cells (Foltz et al., 2018; Zaiatz-Bittencourt et al., 2018). However, it is noteworthy that most immune studies in murine models involved long treatment regimes, which may have limited data on early phase effects of the TME on the NK cells. In addition, we demonstrated that a high dosage of TGFβ could not sustain or increase Eomes nuclear localization, which may mirror long treatment regimens that likely harbor high TGFβ concentrations. This could also partially explain why TGFβ has been viewed as immunosuppressive. Moreover, it is not uncommon for cells to undergo desensitization due to persistently high levels of TGFβ, and that the TGFβR receptor could have been internalized due to high levels of TGFβ (Mitchell et al., 2004; Duan and Derynck, 2019). Furthermore, it was previously reported that high NKG2D ligands in endothelial cells resulted in NKG2D receptor internalization in NK cells (Thompson et al., 2017). Additionally, by super-resolution imaging of NK actin reorganization, we found that only treatment with low dosage of TGFβ showed filopodia-like structure and homogeneous increase in actin intensities (indicative of dynamic RhoA activation). By contrast, higher dosages induced intense local enhancement of actin condensation. It is well known that contractility induced by RhoA is dynamic and spatio-temporally coordinated (Pertz, 2010; Narumiya and Thumkeo, 2018). A persistent and global activation of RhoA with high dosage of TGFβ could have fired aberrant signaling pathways that masked the perceived effects of RhoA-MLC contractility-induced Eomes nuclear localization. Further works are required to understand whether and how RhoA activation is tightly controlled to specifically enhance NK cell contractility for Eomes nuclear localization.

What else could be the trigger for intracellular contractility in NK cells that lack focal adhesions for force transduction? One possibility is the force transmission at the immune synapse/other surfaces. For instance, the KLRG1 receptor on NK cells recognizes E-cadherin on target cells (Nakamura et al., 2009), and it has been demonstrated that E-cadherins adhesive clusters at cell junctions are mechanically coupled to actomyosin cortex to generate junctional tension (Wu et al., 2015). Hence, future work may identify whether an interplay of different biological parameters co-regulate such intracellular contractility in non-adherent immune cells.

Since the nucleus of eukaryotic cells is mechanically coupled to the focal adhesion complex and the actin cytoskeleton by the LINC complex (Guilluy et al., 2014), enhanced intracellular contractility exerts a mechanical force on the nuclear membrane. Such force exertion deforms the nuclear membrane and increases nuclear pore exposure to the cytosolic versus nuclear side of the membrane to favour protein import (Elosegui-Artola et al., 2017). Our results indicate that the NSCLCs induce significant nuclear flattening, which we demonstrated to be a consequence of enhanced contractility. Hence, we propose that such mechanical deformation of the nucleus is recapitulated in mechanosensitive NK cells undergoing anticancer activities; this action facilitates Eomes transcription factor nuclear shuttling. NK cells lack focal adhesion complexes to couple force transmission from ECM to actomyosin complex; therefore, the ability to intrinsically fine-tune its intracellular contractility according to the aggressiveness of cancer cells, and apply the induced mechanoforce for Eomes to gain traction into the nucleus is a novel attribute for translational therapeutics consideration. Future studies may ascertain whether the LINC complex, which couples cytoskeletal force to nuclear pore enlargement, is directly involved in Eomes nuclear entry. In addition to ultimately defining how Eomes can be mechanistically regulated, it would be pertinent in future, to identify pathways that simultaneously engage cytoskeletal remodeling and dynamism. For example, both the activating receptors, NKG2D and DNAM-1, are known to induce actin polymerization through Vav1 (Xiong et al., 2015), which will influence the dynamism of the NK cytoskeletal network. Likewise, the recognition of target cells through β1 and β2 integrins will most likely synergize mechano-responsively in NK cells (Zhang et al., 2020; Santoni et al., 2021).

In conclusion, our results support that Eomes nuclear entry is associated with F-actin re-organization and force-induced nuclear flattening that can be influenced by osmolarity and TGFβ. Our findings offer a vivid mechanism (see Figure 6) on how NK cells attempt to regain cytotoxicity via intracellular contractility which promoted early and prominent accumulation of Eomes (and possibly other mechanosensitive proteins) in the nucleus. Awaiting further exploration is the augmentation of NK cellular contractility though myosin light chain phosphorylation, which could act as a potential “*biological rheostat*” to facilitate activation of dormant NK cytotoxicity against pathophysiological events and prevent excessive metabolic expenditures that may undermine NK activities. Our results also caution researchers that well-studied cytotoxicity promoting TFs in *vitro* investigations may be insufficient to yield highly desirable results *in vivo* when there is a lack of context-dependent understanding of the TFs and cell dynamics. Overall, our findings provide a new avenue for the development of potential immunotherapeutic strategies, for example, through the use of contractility modulating drugs to perform *ex vivo* enhancement of NK cytotoxicity prior to *in vivo* administration in clinical settings.

## Materials and Methods

The general methodologies on cell culture, transfection (plasmids & siRNAs), flow cytometry, ELISA, Western blot, RT-PCR, soft agar assay and antibody list with Resource Reference IDs (RRIDs) are in the “Supplementary Material”.

### Isolation of PBMCs and Primary NK Cells

Apheresis cones from healthy donors were obtained from the Health Sciences Authority Singapore (HSA), under protocols approved by HSA and NUS Institutional Review Board (201706-06 and H-17-028E). Peripheral blood mononuclear cells (PBMCs) were isolated from blood samples using Ficoll-Paque (GE Healthcare, Cat# 17144003) gradient centrifugation. Primary human NK cells were enriched from PBMCs by negative selection using the EasySep Human NK Cell Enrichment Kit (StemCell Technologies, Cat# 19055), following the manufacturer’s protocol.

### NK Cell Coculture With Lung and Breast Cancer Cells

hNK, KHYG-1 and NK-92 cells were co-cultured with NSCLCs: NCI-H1299 or NCI- H1975 at 2.5:1 effector (NK) to target (NSCLC) ratio as was previously determined by the group (Verma et al., 2020). hNK or NK-92 were cocultured with breast cancer cell lines MCF7 or MDA-MB-231 at 2.5:1 effector (NK) to target (breast cancer cell) ratio. Coculture was carried out in NK MACS media supplemented with 25 ng/ml IL2 for primary human NK cells, or 10% RPMI supplemented with 10 ng/ml IL-2 for KHYG-1 and NK-92 cell lines. hNK, KHYG-1 and NK-92 cell lines were pre-stained with CFSE (Sigma), which was able to perpetuate for 6 days as determined by CFSE + population from flow cytometry, following the manufacturer’s protocol for all co-culture setups except for those used for cytotoxicity assay against model target cell line, K562.

### NK Cell Cytotoxicity Assay

Target K562 cells were pre-stained with CFSE prior to coculture with NK cells for cytotoxicity assay. Effector (NK) to target (K562) ratio used was 1:1 for hNK cells and KHYG-1 cells and 3:1 for NK-92 cells. After a 4-h incubation period maintained at 37°C with 5% CO2, cells were stained with fixable viability dye eFluor506 (eBioscience, Cat# 65–0866–14) on ice for 30 min and immediately analysed by flow cytometry for fixable viability dye eFluor506 staining.

### Tonicity Treatment

NK-92 cells were incubated with warm 1x PBS (isotonic), 2X PBS (hypertonic) or 0.5x PBS (hypotonic) for 30 min at 37°C with 5% CO2. Subsequently, NK-92 cells were fixed with warm 4% PFA (Electron Microscopy Sciences, Cat# 15710) for imaging.

### Immunofluorescence Staining and Structured Illumination Microscopy

For immunofluorescence staining, cells were centrifuged and fixed with 4% PFA for 15 min at 37°C. After fixation, free aldehydes were quenched with freshly prepared sodium borohydride (0.01%; Sigma-Aldrich, Cat# 452882) dissolved in 1x PBS for 5 min. Samples were washed thrice with 1x PBS at 5-min intervals and incubated with blocking and permeabilising solution (3% bovine serum albumin and 0.2% Triton X-100 in 1× PBS) for 30 min. After fixation, permeabilization and blocking, samples were incubated with appropriately diluted primary antibodies (1:200 for T-bet and Eomes, and 1: 500 for all other antibodies) in blocking solution for 40 min at room temperature. This was followed by washing thrice with 1× PBS, and incubating with Goat anti-Rabbit IgG (H + L) Highly Cross-Adsorbed Secondary Antibody, Alexa Fluor– 647 conjugated secondary antibodies (ThermoFisher Scientific, Cat# A32733, RRID:AB_2535805), Goat anti-Mouse IgG (H + L) Highly Cross-Adsorbed Secondary Antibody, Alexa Fluor Plus 555) (ThermoFisher Scientific, Cat# A32727, RRID:AB_2633276), and DAPI (ThermoFisher Scientific, Cat# D1306, RRID:AB_2629482)). The cells were washed thrice with 1× PBS. Cells were placed in an iBidi glass-bottom dish (iBidi, Cat# 81218–200) pre-coated with 0.01% poly-l-lysine (Invitrogen, Cat# P8920), and centrifuged at 700 xg for 5 min using a swing-out bucket rotor. The cells were then imaged by confocal or structured illumination microscopy (SIM). For visualisation of actin, Alexa Fluor 488 Phalloidin (ThermoFisher Scientific, Cat# A12379) stain was used together with the secondary antibodies at 1:500 dilution. Imaging was performed on Yokogawa CSU-W1 (Nikon TiE system) using a 1.40 numerical aperture (NA) oil immersion ×100 objective. For SIM imaging, Live-SR module (Roper Scientific) on the spinning disk microscope was engaged during imaging. Post-processing of the images was performed with Live-SR algorithm. For comparison between samples, the binning, laser powers and exposure time were kept constant.

### Laser Ablation

Thirty minutes prior to imaging, suspension NK-92 cells were stained with CellMask™ Actin Tracking Stain (ThermoFisher Scientific, Cat# A57243). The NK-92 cells were then centrifuged onto a PLL coated glass bottom dish at 500 g for 5 min. All subsequent imaging were performed within 30 min after NK-92 cells were centrifuged onto the glass bottom dish to prevent any diminishing effects due to removal of NK-92 cells from cancer cells. To measure recoil velocity due to tensional release of actin network mesh within the cells, distinctive features were monitored before, during and after UV laser ablation. Spatiotemporal information of the distinctive features after UV ablation was obtained and quantified as shown in Figure 3D, using MTrackJ plugin in Fiji ImageJ. Fitting of calculated distance between points of distinctive features was carried out using a linear function and a single exponential function. The single exponential function was used for datasets with slow recoil velocity to obtain slow elastic response of the actin network mesh upon ablation. Next, recoil velocity was calculated as the derivative of the above mentioned functions using methods mentioned in earlier literatures (Meijering et al., 2012; Hara et al., 2016).The linear function is:

$$f\left(t\right)=p_{1}t+p_{2}$$

Where t is the approximate time when UV laser shutter is opened, $p_{1}$ is the deformation speed for linear model, and $p_{2}$ is the initial length between points of distinctive features at t = 0 s for linear model.

The single exponential model is:

$$f\left(t\right)=a+Ae^{\left(\frac{-1\left(t-b\right)}{\;\tau }\right)}$$

Where t is the approximate time when UV laser shutter is opened, a is the initial length between points of distinctive features at t=0 s for exponential model, τ is the ratio of Young’s modulus to viscosity, and A, and b are arbitrary constants.

### TGFβ ELISA

To validate the concentration TGFβ, the supernatants of H1299, H1975 and NK-92 cells alone and after cocultures were harvested after 24 h. Supernatant was diluted and TGFβ was measured using TGF-β1 Human ELISA kit (Invitrogen, Cat# 88-8350-86), according to the manufacturer’s instructions. Briefly, the supernatants were diluted 2-fold with ELISA kit diluent and further diluted 1.4 fold with 1N HCl and 1N NaOH. The supernatant was allowed to incubate overnight with a pre-coated 96 well plate and absorbance readings were measured at 459 and 570 nm.

### Treatment of NK Cells With TGFβ for Contractility Assessment

Recombinant human TGFβ protein (Cat # 240-B-002) was obtained from RnD Systems and diluted at 20 μg/ml in sterile 4 mM HCl containing 1 mg/ml human or bovine serum albumin, according to manufacturer’s protocol. NK-92 cells were treated overnight with TGFβ or control (4 mM HCl containing 1 mg/ml human or bovine serum albumin) before fixing with warm 4% PFA for downstream imaging analysis or lysed with RIPA buffer for western blot analysis.

### TGFβR1 Antibody Blocking

TGFβR1 antibody of various concentrations or IgG isotype control were prepared by diluting in incomplete RPMI media (without serum and antibiotics). NK cells were incubated with the TGFβR1 antibody or IgG isotype control for 90 min at 37°C with 5% CO_2_. Subsequently, NK cells were stimulated with PMA/Ionomycin or human recombinant IL15 for 3 and 4 h, respectively before fixing with warm 4% PFA for imaging.

### Measurement of Nuclear-Cytoplasmic Ratio

Confocal and SIM images obtained were analysed using ImageJ V2.0. The nuclear/cytosolic ratio was analysed as previously described (Elosegui-Artola et al., 2017), with slight modifications to accommodate to the smaller cytoplasmic areas in NK cells. CFSE-stained NK cells were identified in cocultures with lung/breast cancer cells at E (NK): T (cancer cell) ratio of 2.5 : 1. DAPI staining was used to identify the nucleus of NK cells. A Z-stacked image (step size 0.2 μm) encompassing the whole cell was obtained and the stacks demarcated by nuclear DAPI staining were Z-stacked for quantification of nuclear/cytoplasmic ratio. The DIC image, CFSE, DAPI, and T-bet/Eomes channels were then merged to demarcate, respectively, the cell cytoplasm (CFSE and DIC areas without DAPI stain), the nucleus (DAPI) and Eomes/Tbet. To avoid measurement of an ROI (Region on Interest) in the nucleus that exceeded the cytoplasmic ROI, the biggest possible ROI drawn in the cytosol next to the DAPI-stained nucleus was first measured. The same ROI was then shifted inside the nucleus to measure the mean intensity of Eomes/T-bet within the nucleus. The nuclear-cytoplasmic ratio was derived by dividing the nuclear intensity by cytosol intensity.

### Quantification of Intracellular Protein Staining in NK cells

To quantify fluorescence intensity of proteins in NK cells, the cells were first identified as CFSE-positive cells (if NK cells were from cocultures) in confocal imaging. The median fluorescence intensity was quantified from an ROI which resembled the cell shape as demarcated by CFSE/DIC in the 488 nm laser/wide field channel. The ROI was then moved to an area without any cells to measure the median fluorescence intensity of the background. The final median intensity was measured as median fluorescence within the cell bound by the ROI minus that of the background. For quantification of pMLC2, the acquired pMLC2 median fluorescence intensity was divided by the median fluorescence intensity of total MLC2 and presented as ‘Normalized pMLC2 median intensity against Total MLC2’. For the quantification of Eomes median fluorescence intensity, individual values were normalized to the mean value of control condition.

### NSCLC Displacement/Migration Analysis

NCI-H1299 and NCI-H1975 NSCLCs were seeded on iBidi glass bottom dish at 10% confluency for 24 h before imaging. Mitomycin C (Sigma, Cat# M0503) was added to the media 1 h before imaging for cell migratory profiles. Nikon Biostation IMQ was used to capture migratory profiles of H1299 and H1975 NSCLCs using a ×20 objective. Images were captured at 10-min intervals over 8 h with cells incubated at 37 °C with 5% CO_2_. ImageJ (RRID: RRID:SCR_003070) plugin ‘Manual tracking’ was used to capture individual cell movements. Displacement profile was generated using Matlab 2021b with in-house code.

### Statistical Analysis

All graphs are presented as mean *±* SEM. Statistical analysis was carried out using Prism 7.04 (GraphPad Software), with a *p*-value < 0.05 considered as significant. *p < 0.05, **p < 0.01, ***p < 0.001, ****p < 0.0001. Two-tailed Student’s T-tests were used when two cases were compared, and analysis of variance (ANOVA) test was used when more cases were analysed. When data did not meet normality criteria, equivalent non-parametric tests were applied. For all experiments with hNK cells, results were obtained from biological repeats of at least three donors as indicated in the individual legends.

## Data Availability

The original contributions presented in the study are included in the article/Supplementary Material, further inquiries can be directed to the corresponding author, upon reasonable request.
