# Supplementary material for: Lung Cancer Induces NK Cell Contractility and Cytotoxicity Through Transcription Factor Nuclear Localization
Source: Front Cell Dev Biol. 2022 May 16;10:871326. doi: 10.3389/fcell.2022.871326 (PMC9149376; doi:10.3389/fcell.2022.871326)
Supplement: Supplementary file 1 [file Table1.DOCX]

**Supplementary Information**

**Supplementary Figure Legends**

**Figure S1. hNK and NK-92 cells are positive for CD56, T-bet and Eomes.** Flow cytometry analysis of **(A)** The surface expressions of CD3 and CD56 (Quadrant 1; Q1), and intracellular expression of T-bet and Eomes (Quadrant 2; Q2) in isolated primary hNK cells and **(B)** Eomes and T-bet in NK-92 cells. Isolated hNK cells are represented by CD3- CD56+ populations. FMO representations for all flow cytometry antibodies are in Figure S11. **(C)** Figure panels represent the gating strategy to identify CFSE+ and dead NSCLCs after coculture with NK cells. NSCLCs were first identified based on approximate size, singles cells were identified, pre-stained CFSE+ NSCLCs were identified from CFSE- NK cells and dead NSCLCs are represented by FVD506+ population. Spontaneous death was used as a negative control.

**Figure S2. The proliferation of NSCLCs (H1299, H1975) was reduced in coculture with NK cells, and H1299 was more invasive than H1975. (A and B)** H1299 NSCLC control shows higher expression of Ki67 (proliferation marker) compared to that of H1975 NSCLC control. A significant drop in Ki67 expression was observed for both NSCLCs within 1 day of coculture with either (A) NK-92 or (B) hNK cells. hNK caused more prominent drop in Ki67 in NSCLCs. All data are quantified and represented as means ± S.E.M. n=4 for NK-92 cells and n= 6 for hNK. The data are representative of 3 donors for hNK cells. **(C)** The migratory displacement profiles for H1299 (red) and H1975 (blue) are normalized and represented as rose plots. Each cell has 16 trajectories and each line represents one cell trajectory over 8-hour duration. **(D)** H1299 NSCLC has substantially more colonies compared to H1975 NSCLC in soft agar assay. All data are quantified and represented as means ± S.E.M, n= 4; colonies were quantified from 4 independent wells.

**Figure S3. Eomes and T-bet expression remained relatively constant in NK cells cocultured with NSCLCs. (A-D)** The median fluorescence intensity (MFI) Eomes and T-bet proteins were quantified in NK cells using flow cytometry (A and B for hNK, C and D for NK-92). The levels of Eomes and T-bet are relatively constant, with a slight reduction in Eomes expression in hNK cells co-cultured with invasive H1299 NSCLC. All data are quantified and represented as means ± S.E.M, n≥5 and data are representative of 3 donors for hNK cells. **(E and F)** Measurement of Eomes MFI from confocal images showed slight reduction in the normalized Eomes expression in hNK and NK-92 cells coculture with H1299 NSCLC. All samples were stained with a master mix of respective primary and secondary antibodies and imaged with same laser power and exposure time. All data are quantified and represented as means ± S.E.M. n=40 cells for each condition and are representative of 3 donors for hNK cells.

**Figure S4.** **Eomes and T-bet showed similar compartmentalization preference at 2-6 hours of coculture with NSCLCs, and breast cancer cells induced similar Eomes compartmentalization in NK cells. (A and B)** hNK cells showed relatively constant Eomes and T-bet nuclear/cytoplasmic (Nuc/Cyt) localization ratio between control and cocultures with NSCLC. The graphs represent means ± SEM; n≥20 cells for each condition and data are representative of 3 donors for hNK cells. One way ANOVA test was used to compare the means of each group. **(C)** Breast cancer cells (MCF7 and MDA-MB-231) induced an increase in NK-92 Eomes nuclear localization as represented by a higher Eomes Nuc/Cyt ratio. The more metastatic MDA-MB-231 cells induced a higher Nuc/Cyt ratio as early as day 1 of coculture. The graphs represent means ± SEM; n≥35 cells for each condition. One way ANOVA test was used to compare the means of each group.

**Figure S5. T-bet did not undergo prominent nuclear localization when challenged by NSCLCs. (A and B)** With the exception of hNK at day 3 of coculture with H1299, NK-92 and hNK cells showed marginal T-bet nuclear localization preference in NK-NSCLC cocultures. The graphs represent means ± SEM; n≥20 cells for each condition and data are representative of 3 donors for hNK cells. One way ANOVA comparing the means of each group was used. **(C)** Representative SIM images of T-bet in hNK after co-culture with NSCLCs. Red circles demarcate the nucleus of hNK cells as determined by DAPI staining (not shown). Scale bar = 5 μm. **(D)** KHYG Eomes localization increased at day 3 and returned to basal levels similar to control by day 6, n≥22. **(E)** T-bet localization remained relatively constant in KHYG cells co-cultured with NSCLC, n≥15. The graphs are represented as means ± S.E.M.

**Figure S6. Eomes (but not T-bet) promoted cytotoxicity in both NK cell types (NK-92 and KHYG).** **(A-D) Flow cytometry analyses and quantifications show that Eomes overexpression enhanced NK-92 killing of CFSE-labelled target K562 cells. The percentage of dead cells are indicated by FVD506 positive staining.** The graphs are represented as means ± S.E.M, n=4 and n=3 for NK-92 and KHYG, respectively.

**Figure S7. Metastatic invasive NSCLC imbalanced activating and inhibitory receptors. (A-D)** Coculture with NSCLCs progressively reduced expression of both inhibitory (PD-1, NKG2A and TIGIT) and activating (NKG2D) surface receptors in hNK cells. **(E-G)** NSCLCs progressively reduced expression of both the activating and inhibitory surface receptors in NK-92 cells. For all experiments with hNK, n≥6 representative of 3 donors, and for NK-92, n≥4. All graphs are represented as mean ± SEM and unpaired students T-test was used.

**Figure S8.** **NSCLC induced actin polarization and myosin light chain phosphorylation in NK cells. (A)** Line plot quantification of F-actin polarization (related to Figure 3A). Green lines on the cells indicate a cross section of representative image and the line graphs indicate the median intensity of actin across respective cells. **(B)** Representative SIM images of NK-92 cells stained for total MLC and pMLC2, and F-actin was stained with phalloidin. All samples were stained with a master mix of respective primary and secondary antibodies and imaged with same laser power and exposure time. Scale bar = 5 μm. **(C)** Cocultures of NK-92 cells with NSCLCs did not affect total myosin light chain 2 levels in NK-92 cells as meas­ured by the mean staining intensity from immunofluorescence images. Unpaired students T-test was used to compare between conditions. The graph represents means ± SEM, n≥33.

**Figure S9. Tonicity and Rho activator did not affect T-bet localization, and contractility reduced NK-92 surface receptor expression. (A)** NK-92 cells were stained for F-actin with phalloidin, and subsequently hyperstacked using ImageJ program to represent the cell volume. Hypertonic treatment reduced cell volume and increased the available plasma membrane (flatter, indicated by more blue cell). On the other hand, hypotonic treatment increased cell volume and height of NK-92 cells. Scale bar = 5 μm. **(B and C)** Representative images and quantification of T-bet nuclear localisation after 30 minutes of tonicity treatment. Red circles demarcate the nucleus of NK-92 cells. Scale bar = 5 μm. Unpaired students t-test was used, n=16. **(D and E)** Representative images and quantification on the right show no significant differences in T-bet nuclear localisation with Y-27632 and Rho activator treatments. Red circles demarcate the nucleus of NK-92 cells. Scale bar = 5 μm. The graphs represent means ± SEM, n=35. **(F)** Treatment of NK-92 cells with Rho activator (1 μg/ml) or nocodazole (1 μM) reduced the expression of NK-92 surface inhibitory receptors (TIGIT and NKG2A) but did not further increase activating receptor NKG2D, n=3.

**Figure S10. TGFβR1 blocking did not affect Eomes and T-bet protein levels, while H1299 showed higher TGFβ mRNA expression. (A)** Western blot with densitometric quantification (shown below the blots) indicate constant expression of Eomes and T-bet in NK-92 cells treated with anti-TGFβR1 and IgG isotype control antibody. **(B)** TGFβR1 antibody blocking (compared to control IgG), reduced Eomes nuclear localization moderately. The graph represents means ± SEM, n is represented in table next to graph. Scale bar = 5 μm **(C)** No significant difference was observed in T-bet intracellular localization in NK-92 cells treated with IgG control or blocked with anti-TGFβR1 antibody. The graph is represented by means ± SEM and unpaired students T-test was used, n≥47. **(D)** NK-92 cells were CFSE-labelled and flow sorted for total RNA extraction and cDNA synthesis. NSCLCs promote *TGFB1* mRNA production in NK-92 cells. However, only invasive H1299 NSCLC induced significantly higher *TGFBR1* mRNA expression in NK-92 cells (n=3). **(E)**  NK-92 cells were CFSE-stained and analyzed for surface TGFβR expression after coculture with NSCLCs. H1299 NSCLC induces significantly higher surface expression of TGFβR1 proteins on NK-92 cells as determined by flow cytometry, n=7. **(F) The** mRNA expressions of *TGFβ1* and *TGFβR1* in H1299 and H1975 NSCLCs alone or in coculture were analyzed after flow sorting CFSE-labelled NSCLCs from NK-92-NSCLC coculture. H1299 showed higher *TGFB1* mRNA expression, whereas H1975 NSCLCs had higher *TGFBR1* mRNA expression. The graph is represented by means ± SEM and unpaired students T-test was used, n=5. **(G)** NSCLCs were CFSE-stained and analyzed for surface TGFβR1 expression after coculture with NK-92 cells.TGFβR1 surface protein expression as determined by % fold change in MFI relates to observed lesser *TGFBR1* mRNA in (F). The graph is represented by means ± SEM and unpaired students T-test was used, n=4.

**Figure S11. Representative gating strategy for gating of CFSE-labelled NK cells**. **(A)** Figure represents the general gating strategy to differentiate (i) single cells, (ii) live cells (FVD506 negative), (iii) CFSE positive cells and (iv) protein of interest, in this case, surface PD-1 expression (Quadrant 2; Q2) on hNK cells. **(B-M)** Fluorescence Minus One (FMO) representation of conjugated (B-L) and non-conjugated (M) antibodies used for flow cytometry analysis in this research. The unstained, FMO and protein of interest are labelled on the graphs and Y axes are normalized to the mode.

**Supplementary Materials and Methods**

**Cell culture**

Isolated primary human NK (hNK) cells were expanded for one week before experiments. hNK was cultured in NK MACS medium (Miltenyi Biotec) supplemented with 1% NK MACS (Miltenyi Biotec), 5% human AB serum (Sigma-Aldrich) and 25 ng/mL IL-2 (Miltenyi Biotec). NCI-H1299 (RRID:CVCL_0060), NCI-H1975 (RRID:CVCL_1511 ), MCF7 (RRID: CVCL_0031), MDA-MB-231 (RRID: CVCL_0062) and K562 (RRID:CVCL_0004) obtained from the American Type Culture Collection, and KHYG-1 (RRID:CVCL_2976) cell line was obtained from DSMZ-German Collection of Microorganisms and Cell Cultures GmbH. KHYG-1 was maintained in RPMI 1640 (Gibco) supplemented with 10% FBS (HyClone) and 1% penicillin/streptomycin (Invitrogen). NK-92 cell line was maintained in RPMI 1640 supplemented with 12.5% FBS, 12.5% horse serum (Gibco) and 1% penicillin/streptomycin. For the culture of KHYG-1 and NK-92 cell lines, RPMI was supplemented with 10 ng/mL IL-2. For NSCLC and breast cancer cell lines, TrypLE Select Enzyme (Gibco) was used for passaging cells. Only cells within passages 3 to 15 were used for experiments. All cell lines were routinely tested and verified to be free from mycoplasma. All cells were grown at 37°C with 5% CO2.

**Antibodies and Reagents**

**Table S1** lists the antibodies and reagents used for experiments.

**Plasmids and Transfection**

pcDNA3.1(+) vector containing Eomes was purchased from Genscript (#OHu25197, RRID:SCR_002891). T-bet was cloned into pcDNA3.1(+) vector (Invitrogen) using EcoRI and XhoI restriction sites. NK cells were transfected with siRNA or plasmids encoding T-bet or Eomes genes using the Neon Transfection System (Invitrogen) and according to the manufacturer recommended parameters. Following transfection, cells were allowed to recover in antibiotic-free media for 48 hours.

**Flow cytometry**

The antibodies used for flow cytometry analysis are represented in Table S1 and their respective FMO are represented in Figure S11. For cell surface staining of receptors, NKG2D, NKG2A, TIGIT and PD-1 antibodies diluted respectively in 1x PBS supplemented with 2% FBS at 1:100 dilution was used to stain NK cells on ice for 10 minutes, followed by staining with fixable viability dye eFluor506 (eBioscience) on ice for 30 minutes. The cells were immediately sent for flow cytometry analysis to distinguish between live and dead cells, and the expression of surface proteins. For staining of TGFβR1 surface expression, TGFβR1 primary antibody was diluted in 1x PBS supplemented with 2% FBS at 1:100 dilution to stain NK cells on ice for 15 minutes, followed by staining with fixable viability dye eFluor506 (eBioscience) on ice for 30 minutes. The cells were then fixed with Intracellular Fixation and Permeabilization Buffer Set (eBioscience) for 30 minutes and stained with secondary PE antibody for 30 minutes. For detection of intracellular proteins, cells were fixed with Intracellular Fixation and Permeabilization Buffer Set (eBioscience) for 30 minutes. Fixed cells were then stained for 30 minutes with the respective conjugated antibodies in permeabilization buffer at 1:100 dilution. Flow cytometry data was acquired using CytoFLEX LX machine from Beckman Coulter (RRID:SCR_008940), and all flow cytometry data were analysed using FlowJo V10.4 (RRID:SCR_008520).

**Western blot**

The Western blot procedures were performed, as previously described (Pan, M. et al., 2020). The results were visualised by ChemiDoc Touch (Bio-Rad) (RRID:SCR_019037) and analysed using Image Lab V 5.2.1(RRID:SCR_014210).

**RT-PCR detection of mRNA**

RNA extraction was carried out using Pure-NA™ Fast Total RNA extraction kit following the manufacturer’s protocol. The cDNA across samples was generated from equal amounts of RNA using SuperScript IV VILO Master Mix following the manufacturer’s protocol. Diluted cDNA (20 ng/ul) was used to quantify the delta Cq values of the samples using SsoFast™ EvaGreen® Supermix from Biorad on the Bio-Rad CFX 96 Real-Time PCR Detection System (RRID:SCR_018064).

**Soft Agar Assay**

The soft agar assay performed in a 24-well plate consist of two layers of agar gels with the upper layer containing 5000 H1299 or H1975 NSCLCs. The bottom layer contained 2X DMEM in 0.8% agar gel. The middle layer comprised of 5000 NSCLC in 2X DMEM in 0.4% agar gel. 500 μL of 1x DMEM media was added to cover the agar gel. Cells were left to grow in an incubator at 37°C with 5% CO2 and medium was changed every 3 days. After 20 days of culture, cells were fixed with ice cold methanol for 5 minutes and stained with 0.001% crystal violet overnight. Wells were washed three times with double-distilled water at 30-minute intervals each. Cell colonies were imaged with a dissecting microscope and counted using ImageJ plugin.

**Supplementary Table**

**Table S1**

| **Key antibodies and chemicals used in work** | |  |  |  |
| --- | --- | --- | --- | --- |
| **Antibody** | **Catalogue No.** | **Manufacturer** | **RRID ^@^** |  |
| T-bet | 4B10 | eBioscience | RRID:AB_763636 |  |
| Eomes | ab23345 | Abcam | RRID:AB_778267 |  |
| GAPDH | sc-32233 | SantaCruz | RRID:AB_627679 |  |
| TGFβRI Antibody | sc-399 | SantaCruz | RRID:AB_632490 |  |
| Normal rabbit IgG | sc-2027 | SantaCruz | RRID:AB_737197 |  |
| T-bet-PE | 4B10 | eBioscience | RRID:AB_763636 |  |
| Eomes-APC | ab23345 | eBioscience | RRID:AB_778267 |  |
| CD3-eFluor450 | OKT3 | eBioscience | RRID:AB_467056 |  |
| CD56-APC | CMSSB | eBioscience | RRID:AB_1210587 |  |
| PD1-PE | REA1165 | Miltenyi Biotec | RRID:AB_2752074 |  |
| TIGIT-PE | REA1004 | Miltenyi Biotec | RRID:AB_2751347 |  |
| NKG2A-APC | REA110 | Miltenyi Biotec | RRID:AB_2655386 |  |
| NKG2D-PE | BAT221 | Miltenyi Biotec | RRID:AB_871658 |  |
| pMLC2 (Thr18/Ser19) | 3674 | Cell Signaling Technology | RRID:AB_2147464 |  |
| pMLC2 (Ser19) | 3671 | Cell Signaling Technology | RRID:AB_330248 |  |
| MLC2 | 3672 | Cell Signaling Technology | RRID:AB_10692513 |  |
| Perforin-eFluro 450 | 48−9994−42 | Invitrogen | RRID:AB_2574145 |  |
| PE-anti-rabbit antibody | P-2771MP | Invitrogen | RRID:AB_2539845 |  |
| Granzyme B-APC | 372204 | Biolegend | RRID:AB_2687028 |  |
|  |  |  |  |  |
| **Chemicals** | **Catalogue No.** | **Manufacturer** |  |  |
| Rho Activator | CN-03-A | Cytoskeleton, Inc. |  |  |
| Blebbistatin | B-0560 | Sigma-Aldrich |  |  |
| Nocodazole | M1404 | Sigma-Aldrich |  |  |
| Y-27632 | Y0503 | Sigma-Aldrich |  |  |
| Alexa Fluor™ 488 Phalloidin | A12379 | Invitrogen |  |  |
| Human TGFβ | 240-B | R&D Systems |  |  |
| Human IL15 | 170-076-114 | Miltenyi Biotec |  |  |
| Human IL2 | 130-097-745 | Miltenyi Biotec |  |  |

^@^ RRID, Resource Reference ID
