# Supplementary material for: Lung Cancer Induces NK Cell Contractility and Cytotoxicity Through Transcription Factor Nuclear Localization
Source: Front Cell Dev Biol. 2022 May 16;10:871326. doi: 10.3389/fcell.2022.871326 (PMC9149376; doi:10.3389/fcell.2022.871326)

Figure S1. hNK and NK-92 cells are positive for CD56, T-bet and Eomes, and gating strategies for identifying CFSE+ dead NSCLCs from NK-NSCLC coculture.

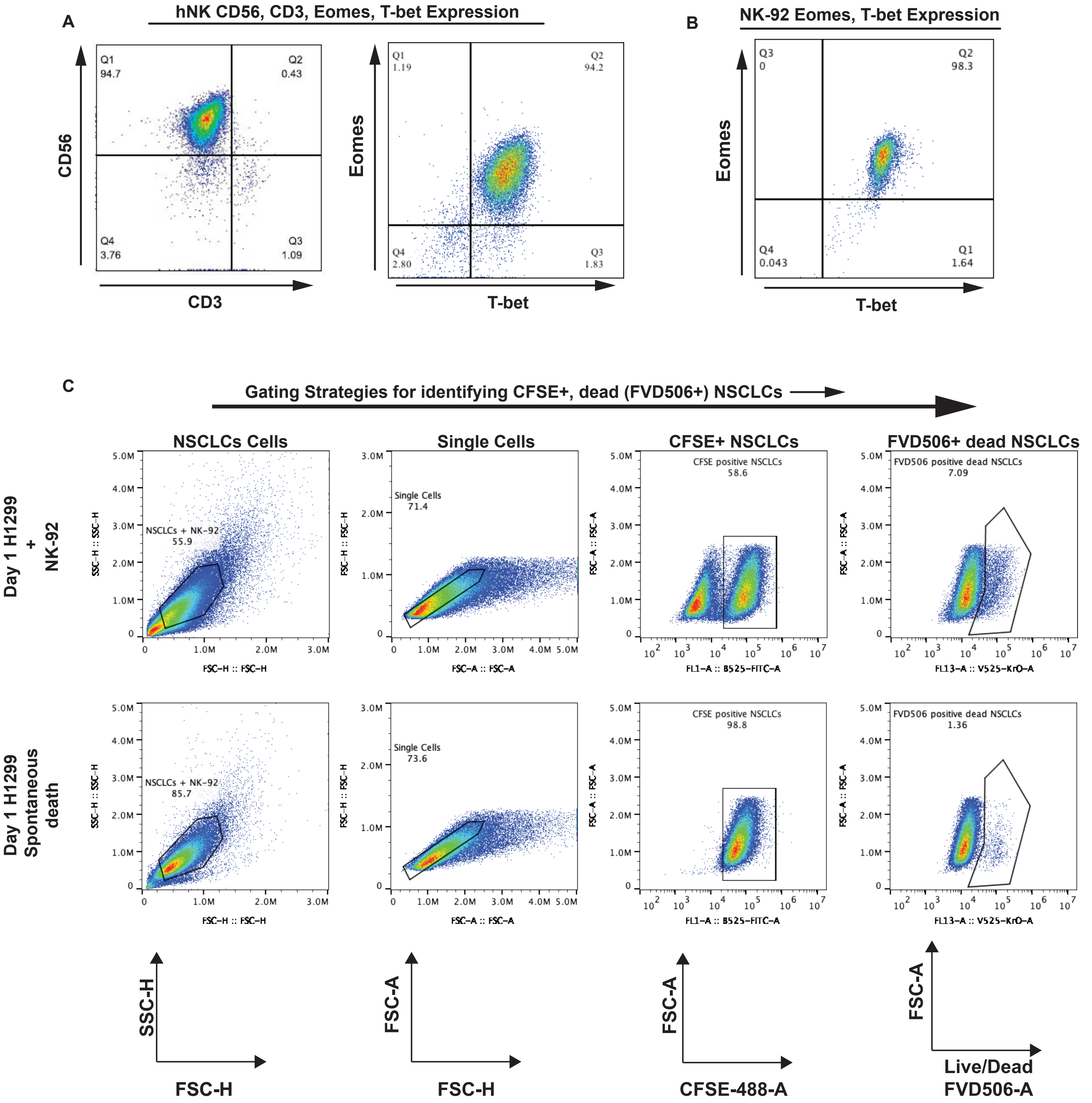

Figure S2. The proliferation of NSCLCs (H1299 , H1975) was reduced in coculture with NK cells, and H1299 was more invasive than H1975.

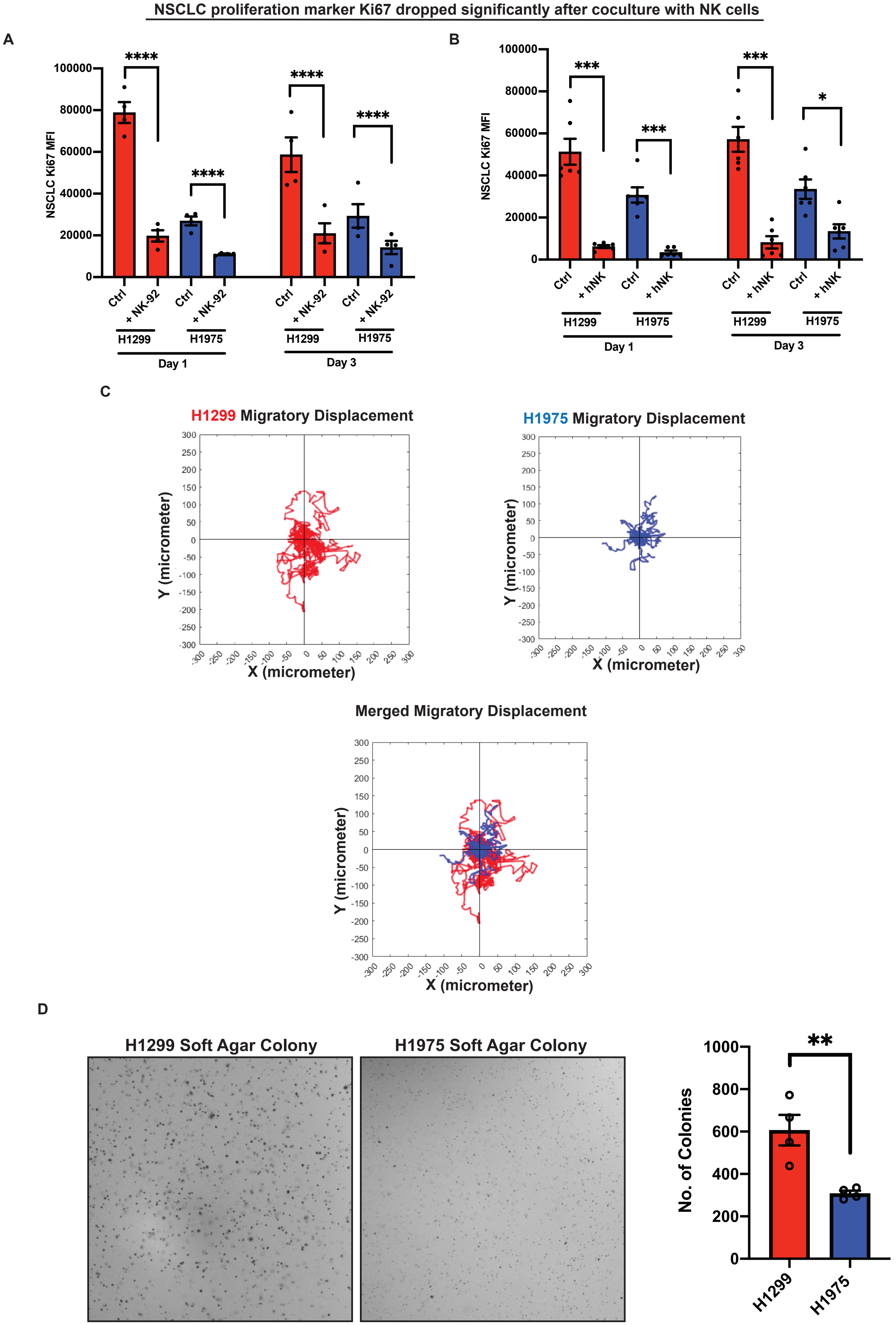

Figure S3. Eomes and T-bet expression remained relatively constant in NK cells cocultured with NSCLCs.

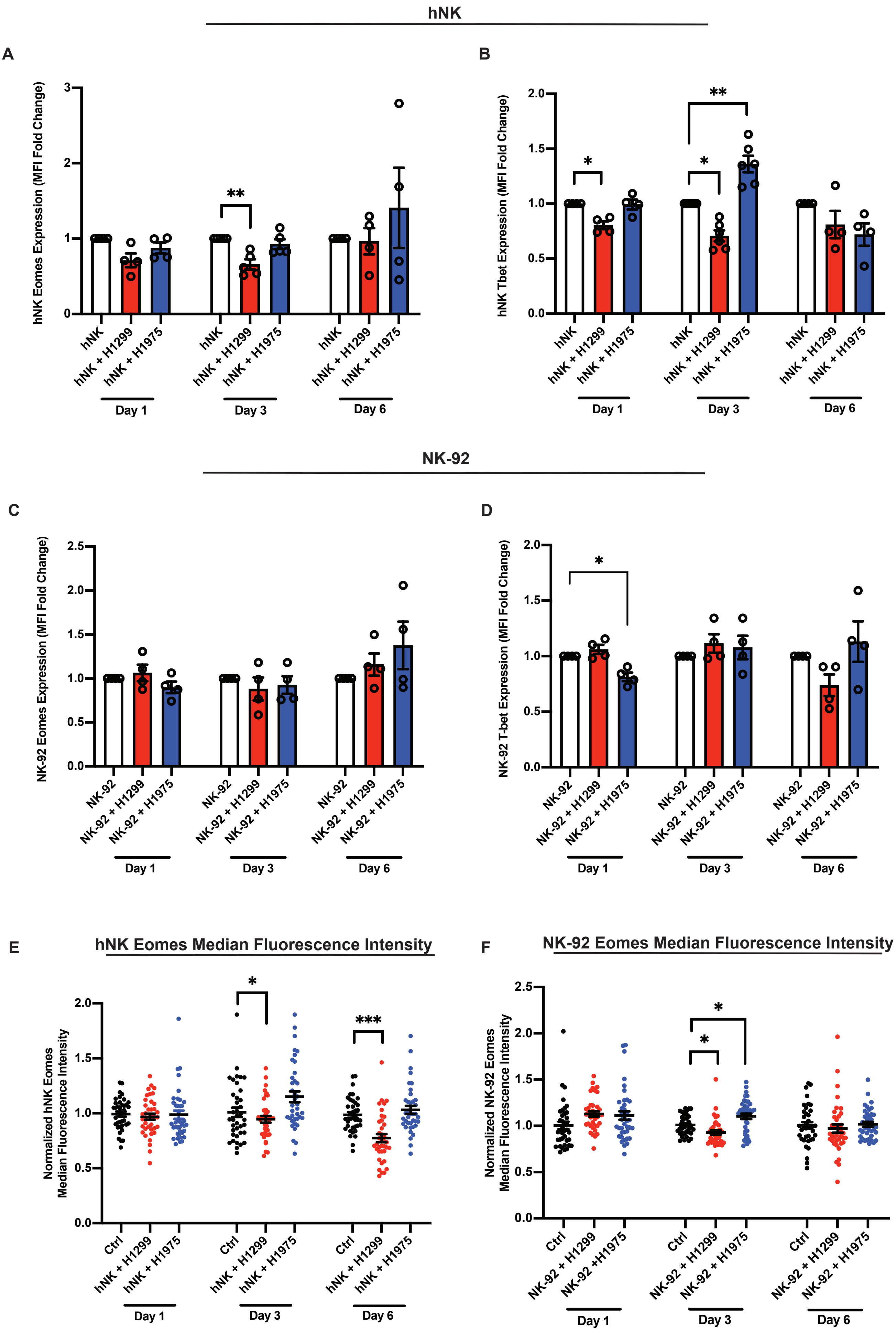

Figure S4. Eomes and T-bet showed similar compartmentalization preference at 2-6 hours of coculture with NSCLCs, and breast cancer cells induced similar Eomes compartmentalization in NK cells.

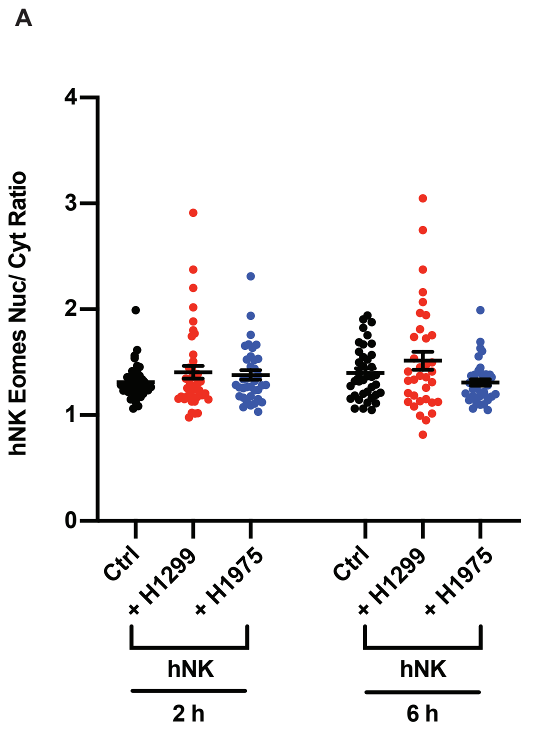

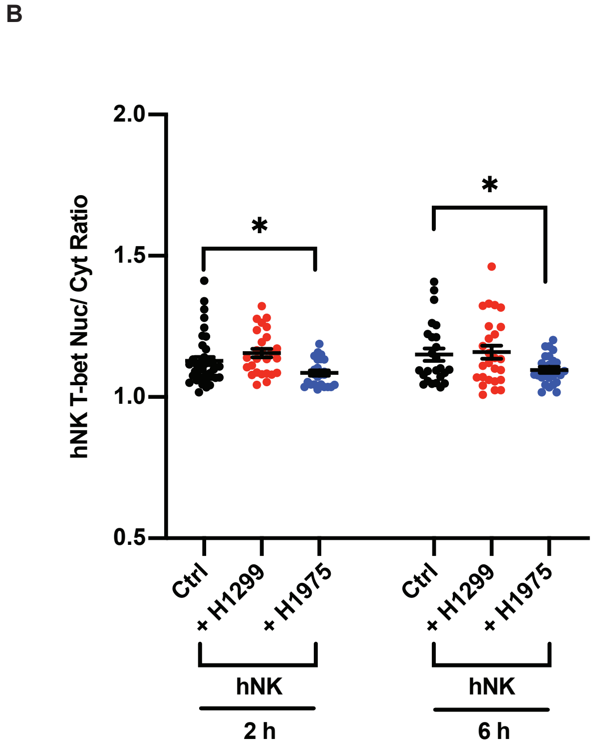

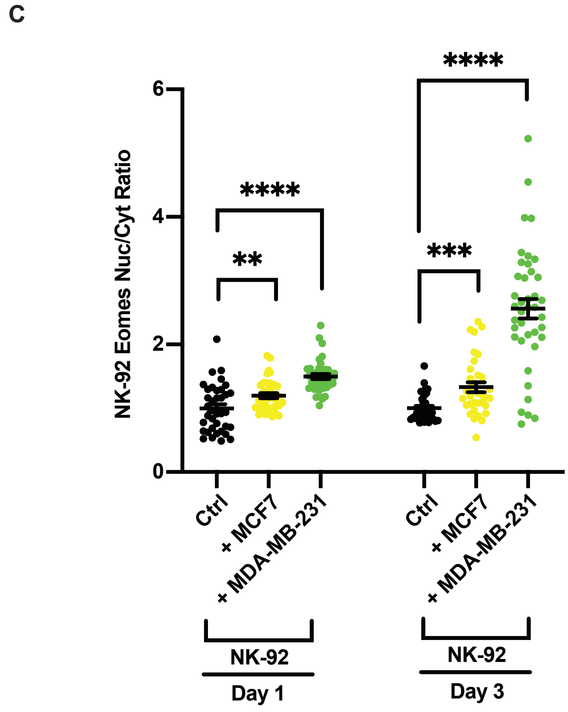

Figure S5. T-bet did not undergo prominent nuclear localization when challenged by NSCLCs.

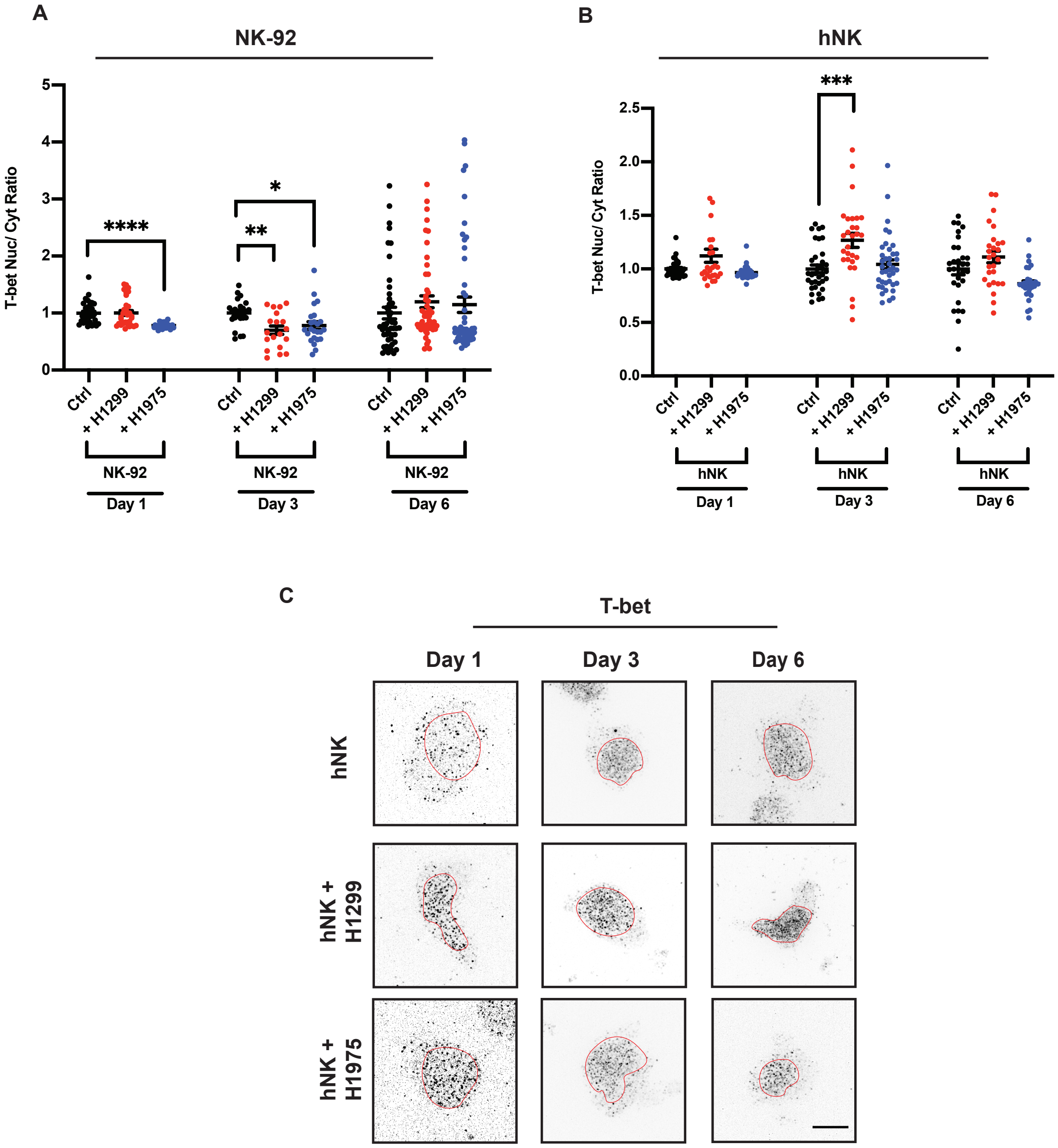

Nuclear localization of Eomes was consistenly more prominent than T-bet in KHYG (± NSCLCs)

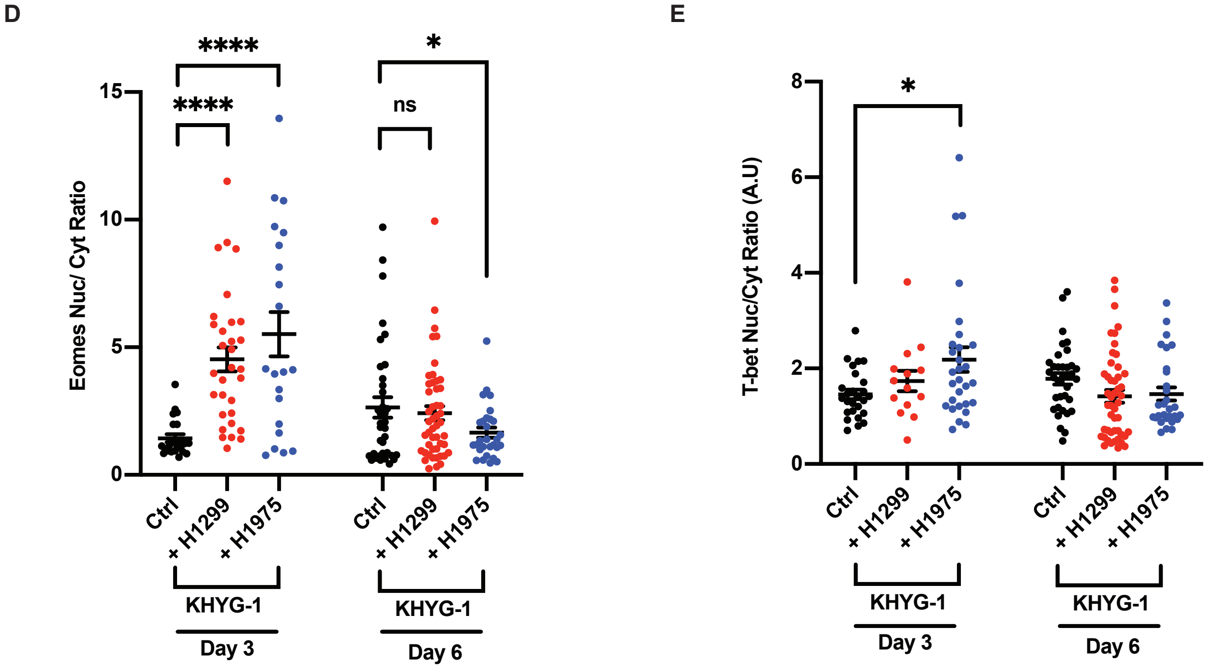

Figure S6. Eomes (but not T-bet) promoted cytotoxicity in both NK cell types (NK-92 and KHYG).

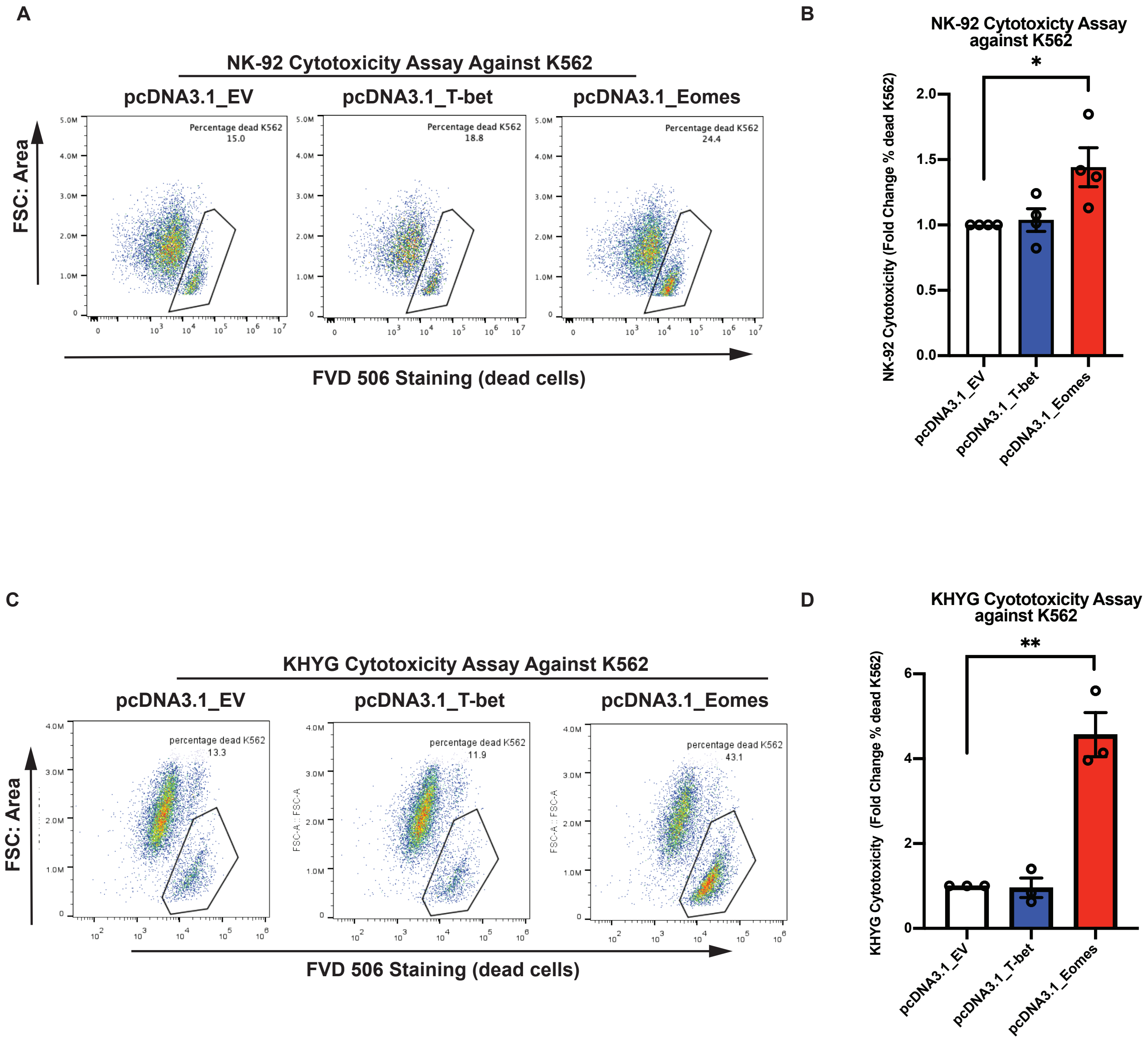

Figure S7. Metastatic invasive NSCLC imbalanced activating and inhibitory receptors.

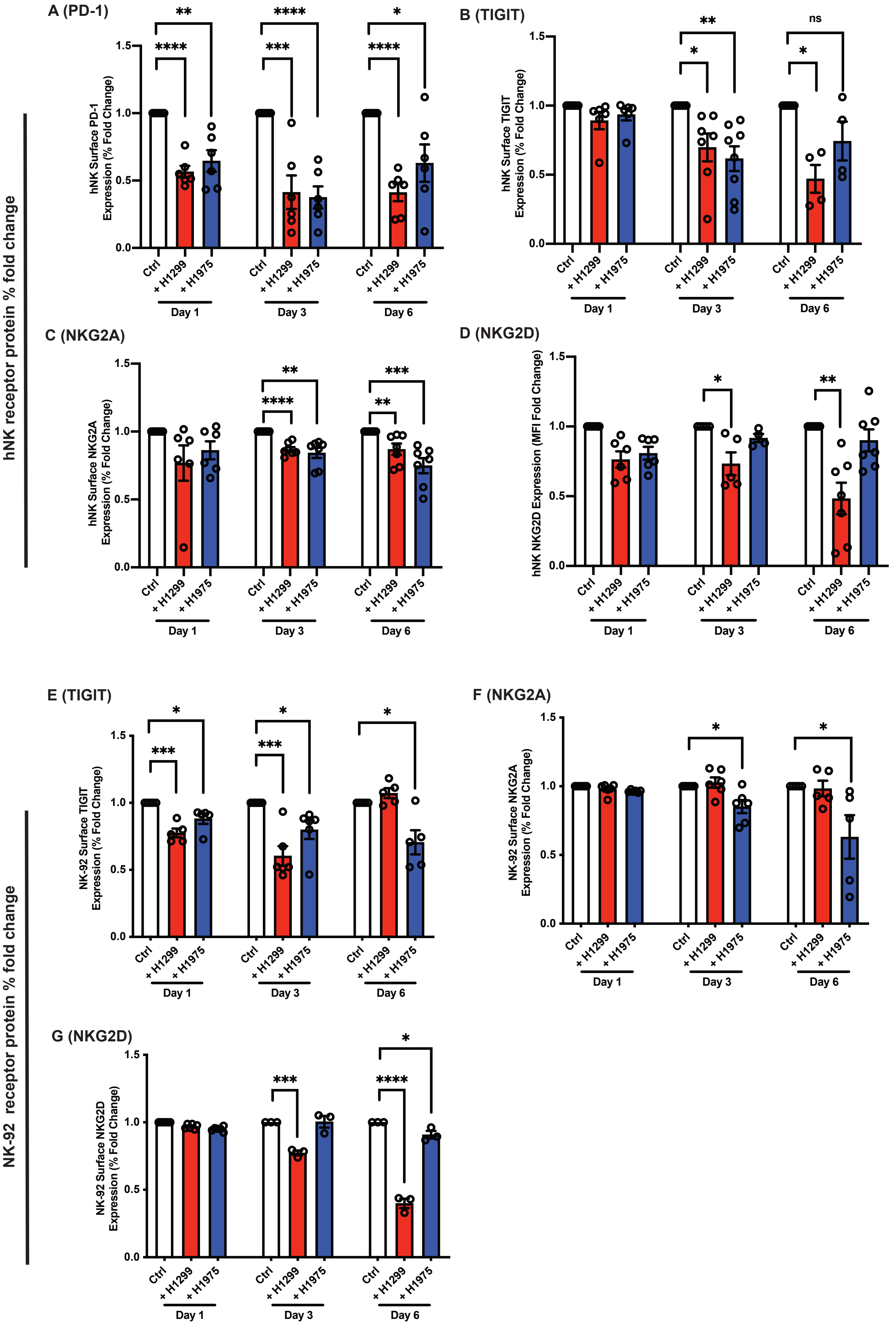

Figure S8. NSCLC induced actin polarization and myosin light chain phosphorylation in NK cells.

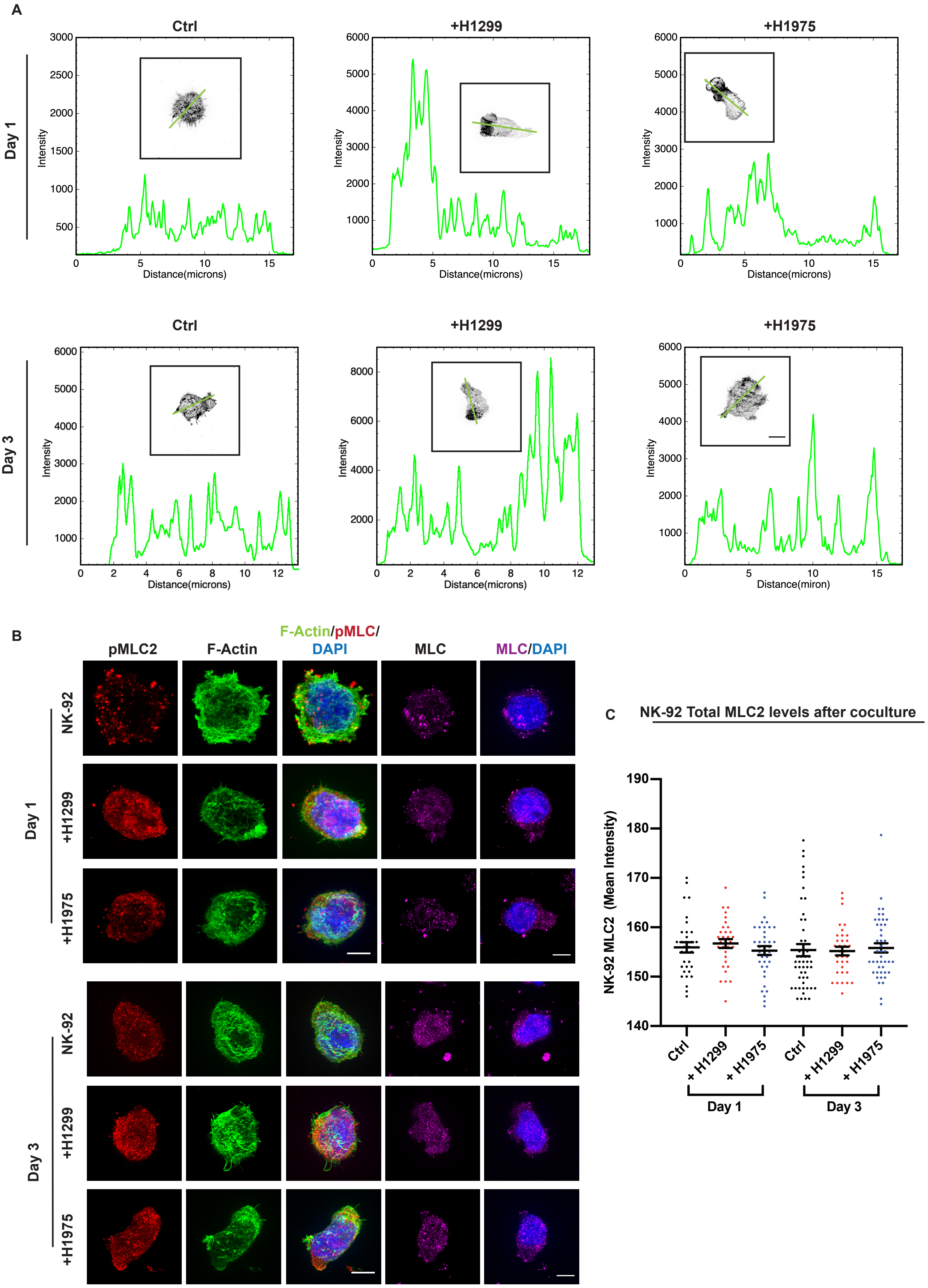

Figure S9. Tonicity and Rho activator did not affect T-bet localization, and contractility reduced NK-92 surface receptor expression.

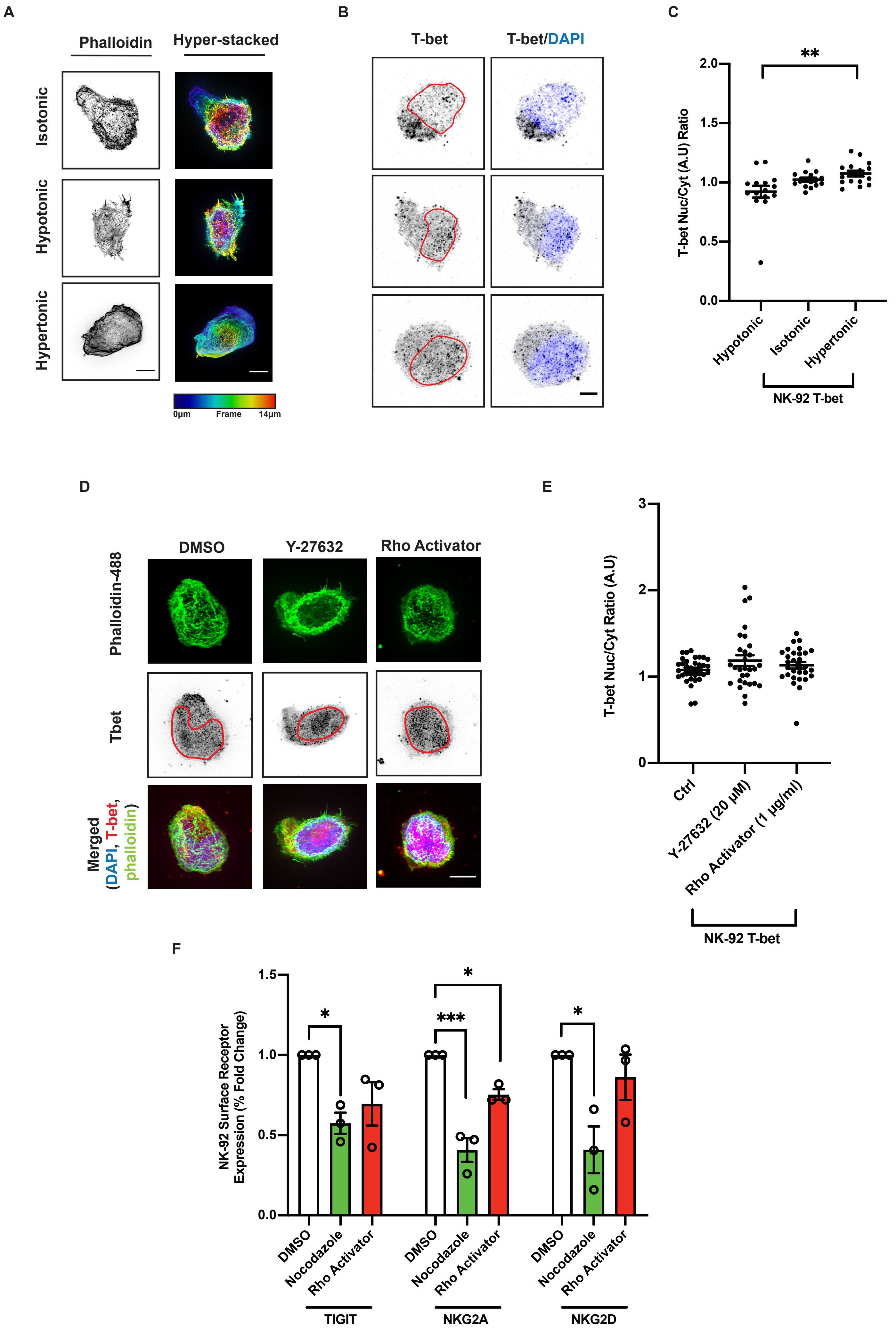

Figure S10. TGFβR1 blocking did not affect Eomes and T-bet protein levels, while H1299 showed higher TGFβ mRNA expression.

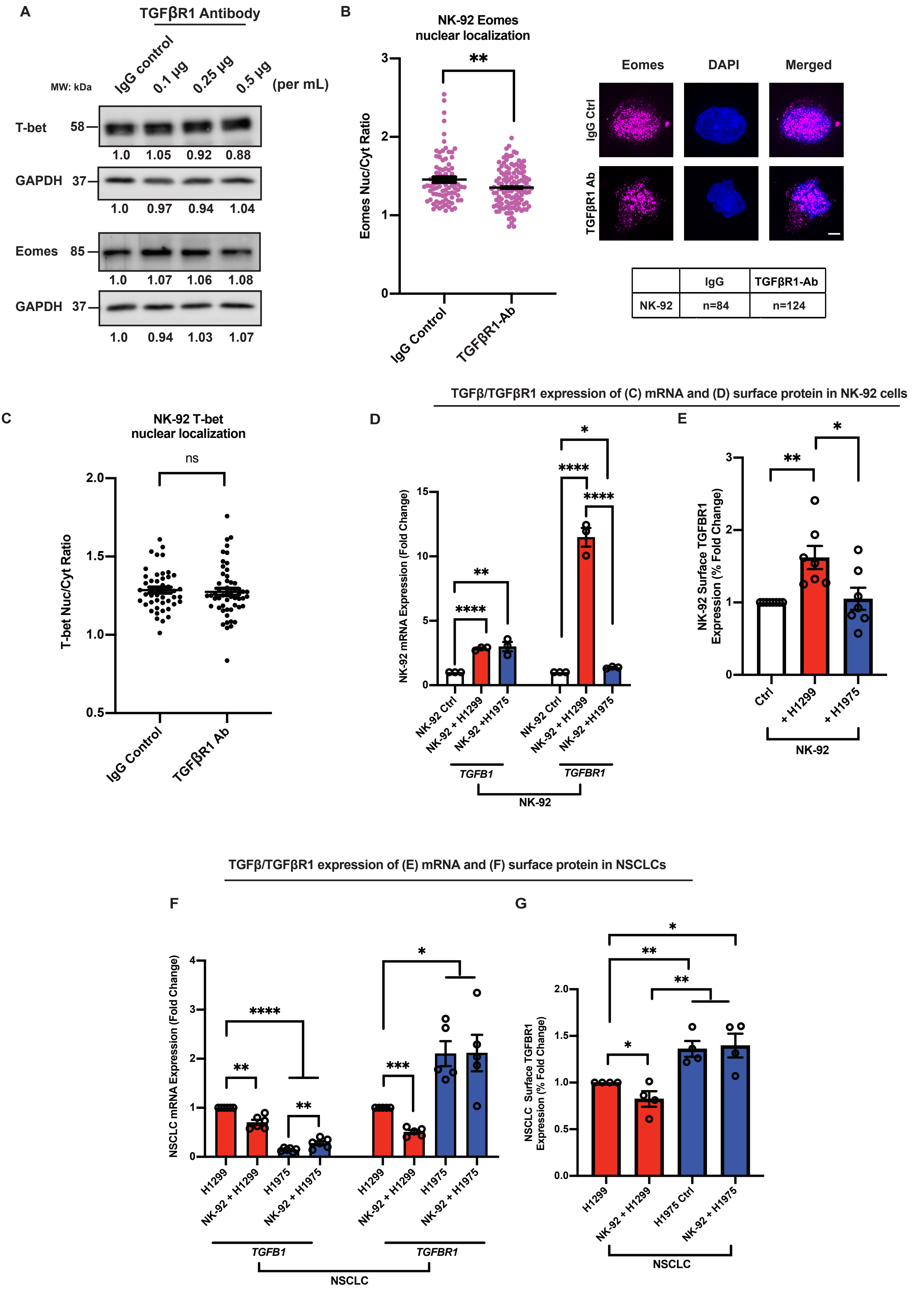

Figure S11. Representative gating strategy for gating of CFSE labelled NK cells and FMO for proteins stained in flow cytometry.

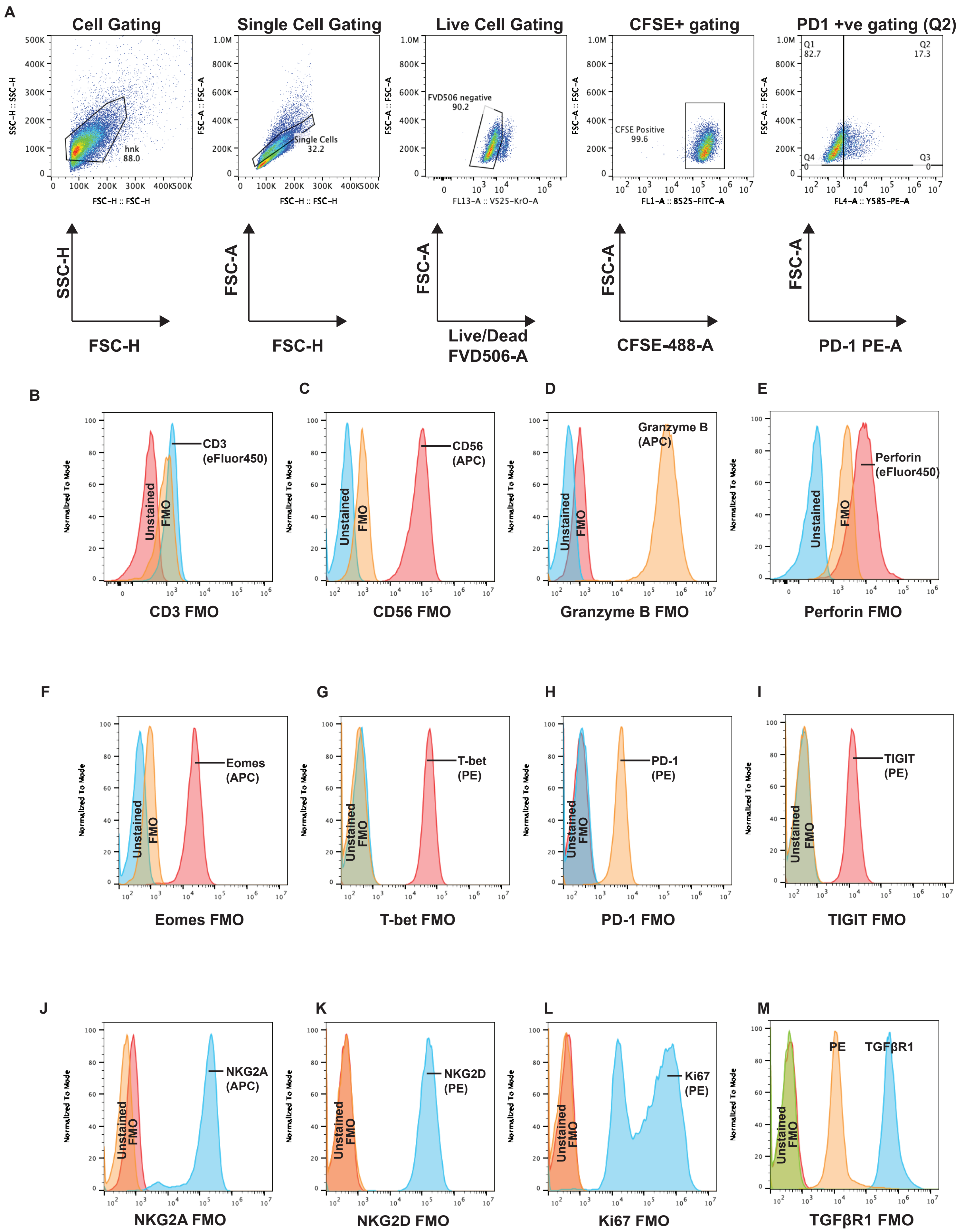

Supplement: Supplementary file 2 [file DataSheet1.PDF]
